# The metalloproteinase ADAM10 requires its activity to sustain surface expression

**DOI:** 10.1007/s00018-020-03507-w

**Published:** 2020-05-05

**Authors:** Anke Seifert, Stefan Düsterhöft, Justyna Wozniak, Chek Z. Koo, Michael G. Tomlinson, Elisa Nuti, Armando Rossello, Doretta Cuffaro, Daniela Yildiz, Andreas Ludwig

**Affiliations:** 1 Institute of Molecular Pharmacology, Medical Faculty, RWTH Aachen University, Aachen, Germany; 2 School of Biosciences, University of Birmingham, Birmingham, UK; 3 Department of Pharmacy, University of Pisa, Pisa, Italy; 4 Institute of Experimental and Clinical Pharmacology and Toxicology, PZMS, ZHMB, Saarland University, Homburg, Germany; 5 Institute of Pharmacology and Toxicology, RWTH Aachen University, Pauwelsstr. 30, 52074 Aachen, Germany

**Keywords:** Metalloproteinase, ADAM10, Extracellular vesicles, Shedding, Inflammation

## Abstract

The metalloproteinase ADAM10 critically contributes to development, inflammation, and cancer and can be controlled by endogenous or synthetic inhibitors. Here, we demonstrate for the first time that loss of proteolytic activity of ADAM10 by either inhibition or loss of function mutations induces removal of the protease from the cell surface and the whole cell. This process is temperature dependent, restricted to mature ADAM10, and associated with an increased internalization, lysosomal degradation, and release of mature ADAM10 in extracellular vesicles. Recovery from this depletion requires de novo synthesis. Functionally, this is reflected by loss and recovery of ADAM10 substrate shedding. Finally, ADAM10 inhibition in mice reduces systemic ADAM10 levels in different tissues. Thus, ADAM10 activity is critically required for its surface expression in vitro and in vivo. These findings are crucial for development of therapeutic ADAM10 inhibition strategies and may showcase a novel, physiologically relevant mechanism of protease removal due to activity loss.

**Electronic supplementary material:**

The online version of this article (10.1007/s00018-020-03507-w) contains supplementary material, which is available to authorized users.

## Introduction

Surface-expressed proteases of the disintegrin and metalloproteinase (ADAM) family function as critical regulators of physiological and pathophysiological processes during development, inflammation, and cancer [1, 2]. ADAM10 is one of the most prominent members of this family. ADAM10 is expressed as a type-1 transmembrane molecule consisting of an N-terminal prodomain, a metalloproteinase domain, a disintegrin domain, a cysteine-rich domain, a transmembrane helix, and finally a cytoplasmic C-terminal region. To a large extent, the function of cell surface-expressed ADAM10 has been attributed to its ability to mediate proteolytic shedding of other surface molecules via its metalloproteinase domain.

ADAM10 can cleave almost 100 different substrates and thus plays critical roles in health and disease [1]. The most important ADAM10 substrate may be Notch controlling cell fate in development [3]. By shedding of Notch family members, ADAM10 controls neuronal differentiation, epidermal integrity, and endothelial angiogenesis [4–6]. However, many more cleavage events have been found to depend on ADAM10 [1]. This includes adhesion molecules of the cadherin family, members of the epidermal growth factor family (EGF and betacellulin), low affinity IgE receptor CD23, ephrins, and inflammatory mediators such as transmembrane chemokines (CXCL16 and CX3CL1) [7]. By regulating these substrates, ADAM10 is involved in endothelial permeability regulation, leukocyte migration [7, 8], transactivation of cancer cells [9], fibrogenesis, immune responses, and infectious diseases [10, 11].

Since ADAM10 fulfills critical functions in inflammatory as well as proliferative pathologies, it is thought that there exist several mechanisms for upregulating or downregulating ADAM10 activity. Following regulation at the transcriptional and translational level, the synthesized protease is converted from a proform into mature ADAM10 by proteolytic removal of the prodomain. On the cell surface interaction with adapter molecules, conformational changes within the cysteine-rich domain or positioning of the ectodomain in relation to the cell membrane can promote binding of the metalloproteinase domain to the cleavage site of the substrate [12]. Moreover, ADAM10 activity on the cell surface may be downregulated by internalization and proteolytic degradation of the protease [13]. Alternatively, ADAM10 itself can undergo shedding or is released in extracellular vesicles [14, 15]. Finally, endogenous inhibitors such as the tissue inhibitor of metalloproteinases (TIMP)-1 can block the activity of the protease [16].

Synthetic small molecule inhibitors of ADAM10 have been tested for their potential to block inflammatory or proliferative events. Such inhibitors were identified among the group of hydroxamate-based pseudopeptide metalloproteinase inhibitors [17]. These inhibitors bind to the protease via residues of their pseudopeptide backbone and coordinate the zinc ion in the active site of the proteases via their hydroxamate group. In previous studies, we have identified and characterized the reverse hydroxamate inhibitor GI254023X (in the following abbreviated as GI) as potent and preferential inhibitor of ADAM10-mediated shedding events [18]. By measuring the activity of the recombinant metalloproteinase domains of ADAM10 or ADAM17, we showed that the inhibitor has a 100-fold higher potency to block ADAM10 than ADAM17. Structural data indicate that the S1 binding pocket of ADAM10 possesses an extended cavity, which is absent in ADAM17 [19] and hence, the large phenylpropyl residue of GI in the P1′ position would fit in the S1′ pocket of ADAM10 but not in that of ADAM17 [18]. Consistent with these findings, the inhibitor effectively blocks ADAM10-mediated ligand-induced shedding of Notch, constitutive and ionomycin-induced shedding of the low affinity IgE receptor CD23, cadherins (N,E, VE) transmembrane chemokines (CXCL16 and CX3CL1) and growth factors (EGF and betacellulin) [7, 20–26]. Recent studies also showed that the inhibitor can be used to block shedding events in vivo and prevent inflammatory processes and fibrosis development in lungs and kidney, respectively [10, 11].

In most previous studies, GI-mediated effects were investigated for only a short period of time which is required for shedding. It is clear that only a few minutes are sufficient for GI to interact with the majority of ADAM10 and that the binding to the active site is likely causing immediate inhibition of ADAM10 activity. However, the long-term treatment effects on ADAM10 over a period of several hours or even days remain unknown. Such knowledge would not only be vital to develop effective ADAM10 inhibition strategies but also allow further important insight in the biology of ADAM10. Here, we studied the long-term effects of GI exposure on surface-expressed and total ADAM10. Surprisingly, we observed that GI exposure led to downregulation of mature ADAM10 which was not due to decreased ADAM10 synthesis but coincided with ADAM10 release in extracellular vesicles. This depletion of ADAM10 resulted in long-term suppression of cellular ADAM10 activity. After removal of GI, the loss of ADAM10 surface activity could only be overcome by de novo synthesis of the protease. The depletion of ADAM10 was not restricted to GI or related hydroxamate inhibitors but was also seen with the natural inhibitor TIMP-1, suggesting that this is a physiological phenomenon. Moreover, mutational inactivation of ADAM10 reduced its surface expression and GI could not further downregulate the inactive protease underlining the importance of ADAM10 activity for its surface expression. Finally, intraperitoneal (i.p.) administration of GI in mice also led to sustained ADAM10 downregulation indicating in vivo relevance of this mechanism. To our knowledge, this is the first report showing that activity of a protease is required to maintain full surface expression and this mechanism may have broader pharmacological implications also for other proteases.

## Materials and methods

### *Antibodies, recombinant proteins*,* and chemical compounds*

Mouse monoclonal antibodies against the N-terminus of human ADAM10 (MAB1427), human ADAM17 (MAB9301), human ERK1/2 (MAB1576), mouse IgG1 isotype control (MAB002), mouse IgG2b isotype control (MAB004), rat monoclonal antibody against the N-terminus of murine ADAM10 (MAB946), and rat IgG2a isotype control (MAB006) were from R&D Systems (Wiesbaden, Germany). Mouse monoclonal antibodies against human/murine GAPDH (MA5-15738), human CD9 (10626D) and rat monoclonal eFluor 450-conjugated CD11b antibody (48-0112-82) were from Thermo Fisher Scientific (Waltham, USA). Allophycocyanin (APC)-conjugated goat anti-mouse (115-135-164), phycoerythrin (PE)-conjugated goat anti-rat (112-116-071), horseradish peroxidase (HRP)-conjugated goat anti-mouse, goat anti-rabbit and goat anti-rat antibodies were from Jackson ImmunoResearch Laboratories, Inc. (West Grove, USA). Rabbit polyclonal antibodies against the C-terminus of ADAM17 (AB19027) and ADAM10 (AB19026) were from Merck Millipore (Darmstadt, Germany). Rabbit polyclonal anti-human HSP70 antibody (EXOAB-Hsp70A-1) was from System Biosciences (Palo Alto, USA). Mouse monoclonal antibody to flotillin-1 (610821) was from BD Biosciences (Heidelberg, Germany). FITC-conjugated rat monoclonal anti-mouse F4/80 antibody (MCA497F) was from Serotec (Bio-Rad, Hercules, USA). The metalloproteinase inhibitors GI254023X, MN8, and MN8-Cy5.5 (CAM36) were synthesized and characterized as preferential ADAM10 inhibitors as previously reported [18, 21, 27]. TAPI-1 was from Merck (Darmstadt, Germany). NH_4_Cl and saponin were from Carl Roth (Karlsruhe, Germany), monensin was from Tocris Bioscience (Bristol, UK), cycloheximide, ionomycin, and bafilomycin A1 were from Sigma-Aldrich (St. Louis, USA), ikarugamycin was from biomol (Hamburg, Germany), recombinant human TIMP-1 (970-TM-010) was from R&D Systems (Minneapolis, USA), *E. coli* pHrodo (P35366), EZ-Link™ Sulfo-NHS-LC-Biotin, and Aldehyde/Sulfate latex beads were from Thermo Fisher Scientific (Waltham, USA), Streptavidin-Sepharose High Performance was from GE Healthcare (Chicago, USA) and the ADAM10 substrate peptide (PRYEAYKMG) was from peptides & elephants (Hennigsdorf, Germany).

### Cell culture, transduction, and transfection

The human monocytic leukemia cell line THP-1 was cultured in RPMI medium with 10% FCS (PanBiotech, Aidenbach, Germany) and 1% penicillin–streptomycin (Sigma-Aldrich, St. Louis, USA). The alveolar lung carcinoma epithelial cell line A549, the human embryonic kidney cell line HEK293, and the murine macrophage cell line RAW264.7 were cultured in Dulbecco’s Modified Eagle’s Medium (DMEM) with 10% FCS and 1% penicillin–streptomycin. Human neutrophils from healthy donors and murine BMDMs were isolated and cultured as described [28]. Experiments with human neutrophils were approved by the local ethical committee of the RWTH Aachen Medical Faculty (EK 259/13) and conform the declaration of Helsinki. ADAM10-deficient HEK293 cells were generated by CRISPR/Cas 9 technique and kindly provided by Björn Rabe, University of Kiel [29].

Short hairpin RNA (shRNA) targeting ADAM10 was inserted into the lentiviral expression vector pLVTHM as described [30]. For expression of shRNA, recombinant lentiviruses were produced as described [30]. For transduction, 2 × 10^5^ THP-1 cells were seeded into 12-wells and concentrated lentivirus preparation (5 µl) was added. To enhance the transduction efficiency polybrene (4 µg/ml, Sigma-Aldrich, St. Louis, USA) was used.

For overexpression of murine ADAM10, coding DNA was inserted into the pMOWS system by usage of the NEBuilder HiFi DNA Assembly Cloning Kit from New England BioLabs (Frankfurt, Germany) and transiently transfected into ADAM10-deficient HEK293 cells using Lipofectamine 3000 (Thermo Fisher Scientific, Waltham, USA).To generate the E/A mutant, the GAA codon at position 1376–78 of murine ADAM10 cDNA was replaced by GCA with the CloneAmp HiFi PCR Premix from Takara (Takara Bio Europe, St-Germain-en-Laye, France). To generate the HE switch mutant, the GAA codon at position 1376-78 and the CAT codon at position 1385-87 of murine ADAM10 cDNA were exchanged.

### Cell treatment

For treatment with inhibitors, cells were seeded in fully supplemented medium with GI254023X (10 µM), TAPI-1 (10 µM) or 0.1% dimethylsulfoxide (DMSO). If not stated differently, the treatment time was 24 h. To analyze the recovery of ADAM10, cells were washed with PBS and received new complete growth medium for up to 24 h. For analysis of cellular protein content, cell lysates were generated using a lysis buffer containing 20 mM Tris–HCl, 150 mM NaCl, 1% TritonX-100, 5 mM EDTA, 1 mM PMSF, 10 mM 1,10-phenanthroline monohydrate, onefold Complete (Roche) and 10 µM GI. After 20 min incubation on ice, lysates were cleared by 10 min centrifugation at 16,100 × *g* and analyzed via a BCA assay (Interchim, Montluçon, France) according to manufacturer's instructions. For flow cytometric analysis of ADAM10 and ADAM17, surface expression adherent cells were harvested using accutase (Sigma-Aldrich, Steinheim, Germany).

### Flow cytometric analysis

PBS supplemented with 0.2% BSA was used as assay buffer, and all steps of the staining process were performed at 4 °C. Cells or precipitates of extracellular vesicles and latex beads were stained with primary antibody or appropriate isotype control for 45–60 min on ice. Detection of bound antibodies was performed using APC- or PE-conjugated secondary antibody. For permeabilization, cells were fixed with 2% PFA and afterwards in all steps, 0.1% saponin was added. The fluorescence signal was analyzed by flow cytometry (LSRFortessa, BD Biosciences, Heidelberg, Germany) and evaluated with FlowJo V10 software. Binding of MN8-Cy5.5 to surface-expressed ADAM10 was determined by staining cells with 10 µM inhibitor, washing and subsequent flow cytometric analysis. The unspecific signal which was determined by preincubation with 10 µM GI as a competitor prior to staining with 10 µM MN8-Cy5.5 was subtracted from the obtained fluorescence signal.

### Western blotting

Samples from cell lysates (20 µg total protein), precipitation of surface proteins or vesicle preparations (see below) were heated in reducing or non-reducing SDS-sample buffer (250 mM Tris HCl (pH 6.8), 50% (w/v) glycerol, 10% (w/v) SDS, 0.1% bromophenol blue with or without 5% β-mercaptoethanol) and subjected to SDS–polyacrylamide gel electrophoresis using 10% Tris–glycine gels. Proteins were transferred onto polyvinylidene difluoride membranes (Hybond-P, Amersham). Membranes were blocked with 5% (w/v) non-fat dry milk in PBS with 0.05% Tween for 1 h and probed with primary antibodies against ADAM10 (0.5 µg/ml) or marker proteins for 1 h at room temperature or overnight at 4 °C followed by incubation with HRP-coupled secondary antibodies (diluted 1:30,000) for 1 h. After addition of enhanced chemiluminescence substrate (ECL Prime, GE Healthcare), signals were recorded using the LAS 3000 Image Analyzer^®^ (Fujifilm, Tokyo, Japan) and quantified using the open source ImageJ software (developed by Wayne Rasband, NIH). ADAM10 migrates faster under non-reducing conditions than under reducing conditions. Therefore, detection by N-terminal ADAM10 antibody which requires non-reducing conditions, results in a 55 kDa band whereas the C-terminal ADAM10 antibody detects mature ADAM10 at 70 kDa under reducing conditions.

### Surface biotinylation

To biotinylate surface proteins, the cells were washed three times with ice-cold PBS and incubated for 30 min at 4 °C with EZ-Link Sulfo-NHS-LC-Biotin (1 mg/ml in PBS, pH 8.0, Thermo Fisher Scientific, Waltham, USA). The biotin reagent was quenched by washing the cells three times with 100 mM glycine in PBS. Cells were lysed as described above and 90% of the lysates were incubated overnight with streptavidin–sepharose high performance beads (GE Healthcare) at 4 °C on a rotary mixer to isolate biotin-labeled proteins. The sepharose beads were spun down and washed three times with lysis buffer. Finally, the beads were resuspended in reducing (C-terminal ADAM10) or non-reducing (N-terminal ADAM10) SDS-sample buffer (2 ×) and analyzed via western blot.

### Extracellular vesicle preparation

Extracellular vesicles were prepared as described previously [31]. 20 × 10^6^ THP-1 or A549 cells were cultured in serum-free medium for 24 h. Extracellular vesicles were prepared from supernatants by centrifugation for 10 min at 300×*g*, followed by 20 min at 2000×*g* and 30 min at 10,000×*g*. The resulting supernatant was filtered through a 0.22 µm membrane and extracellular vesicles were sedimented at 100,000×*g* for 75 min at 4 °C using a Beckman rotor Type Ti70. The sediment was resuspended in a high volume of ice-cold PBS and sedimented again at 100,000×*g* for 75 min. For western blot analysis, vesicles were directly dissolved in SDS-sample buffer. For flow cytometric analysis, vesicles were resuspended in PBS and incubated with 50 µl aldehyde/sulphate latex beads (4% w/v, 3.8 µm diameter) overnight at 4 °C. Subsequently, the reaction was stopped by addition of 100 mM glycine. After washing twice, bead conjugates were subjected to flow cytometry as described above.

### Substrate cleavage

ADAM10 activity on THP-1 cells was studied in terms of CXCL16 release. 2.5 × 10^5^ THP-1 cells were pretreated for 24 h with 10 µM GI or vehicle, washed twice with PBS, incubated in fresh growth medium for another 24 h with or without GI and stimulated to express CXCL16 by incubation with *E. coli* pHrodo (THP-1 to *E. coli* ratio is 1:5). Conditioned media were cleared by centrifugation (5 min, 16,100×*g*, 4 °C) and soluble CXCL16 was quantified per ELISA as described [23]. The substrate cleavage assay using betacellulin N-terminally tagged with alkaline phosphatase (AP) has been described previously [32]. HEK293 cells were transfected with plasmid encoding the betacellulin-AP fusion protein by usage of Lipofectamine 3000 (Thermo Fisher Scientific, Waltham, USA). 48 h after transfection, the cells were washed and treated for 45 min with 1 µM ionomycin in DMEM without supplements. Subsequently, AP activity was determined over time in the prepared cell lysates and supernatants by adding a *p*-Nitrophenyl phosphate (PNPP) solution (Thermo Fisher Scientific, Waltham, USA). The AP activity was continuously measured at 405 nm with the FLUOstar Optima. To assess the AP activity, the slope (change of absorption at 405 nm per min) was calculated. The amount of ADAM10 activity was calculated as PNPP substrate turnover (AP activity) in the supernatant in relation to the total turnover in supernatant plus cell lysate.

### Quantitative PCR analysis

The mRNA expression level of ADAM10 was measured by quantitative real-time PCR and normalized to the mRNA expression level of glyceraldehyde-3-phosphate dehydrogenase (GAPDH). RNA was extracted using RNeasy Kit (Qiagen, Hilden, Germany) and quantified photometrically (NanoDrop, Peqlab, Erlangen, Germany). 300 µg RNA was reverse transcribed using PrimeScript™ RT Reagent Kit (Takara Bio Europe, St-Germain-en-Laye, France) and PCR reactions were performed using SYBR Premix Ex Taq II (Takara Bio Europe) according to the manufacturer's protocol. The following primers were used: *ADAM10* forward, ggattgtggctcattggtgggca; *ADAM10* reverse, actctctcggggccgctgac; *GAPDH* forward, ccagccccagcgtcaaaggtg; *GAPDH* reverse, cggggctctccagaacatcatcc. All PCR reactions were run on a LightCycler 480 System (Roche, Basel, Schweiz) with 45 cycles of 10 s denaturation at 95 °C, followed by 30 s annealing at 61 °C (*ADAM10*), or 66 °C (*GAPDH*) and 15 s amplification at 72 °C. Standard curves were determined using a serially diluted internal standard. Relative quantification was performed with the E-Method from Roche Applied Bioscience using the LightCycler^®^480 software 1.5.

### Mice

Animal experiments were approved by the local authorities and performed with 19–21 g male mice (Janvier Labs) on a C57BL/6JRj background (84-02.04.2014.A512; LANUV NRW). Mice received a daily intraperitoneal (i.p.) injection of 200 mg/kg bodyweight GI254023X (300 mM) or DMSO as control over 13 days. Before mice were sacrificed, blood samples were taken retro-orbitally. Lung and liver samples were taken and stored at − 80 °C. For flow cytometric analysis, organ samples were homogenized on ice and erythrocytes were lysed in all samples. Afterwards, cells were stained and subjected to flow cytometry as described above.

### Statistics

Quantitative data are shown as mean and standard deviation (SD) calculated from at least three independent experiments. For a better graphical presentation and comparison flow cytometry, data were normalized by expression in relation to the appropriate controls. Raw data were analyzed by general mixed model analysis (PROC GLIMMIX, SAS 9.4, SAS Institute Inc., Cary, USA) and assumed to be derived from either normal, log normal or beta (percentage data) distributions; residual plots and the Shapiro–Wilk test were used as diagnostics. If necessary, the day of experiment conduction was set as random to assess differences in the size of treatment effects across the results. According to the covtest statement, all data sets were homoscedastic. Multiple comparisons were corrected by false discovery rate (FDR). Significant differences compared to the respective control were indicated as asterisks (*p < 0.05, **p < 0.01, ***p < 0.001). In case of additional comparisons, these were specified by bars.

## Results

### The metalloproteinase inhibitor GI254023X reduces surface-detectable ADAM10

**Fig. 1:**
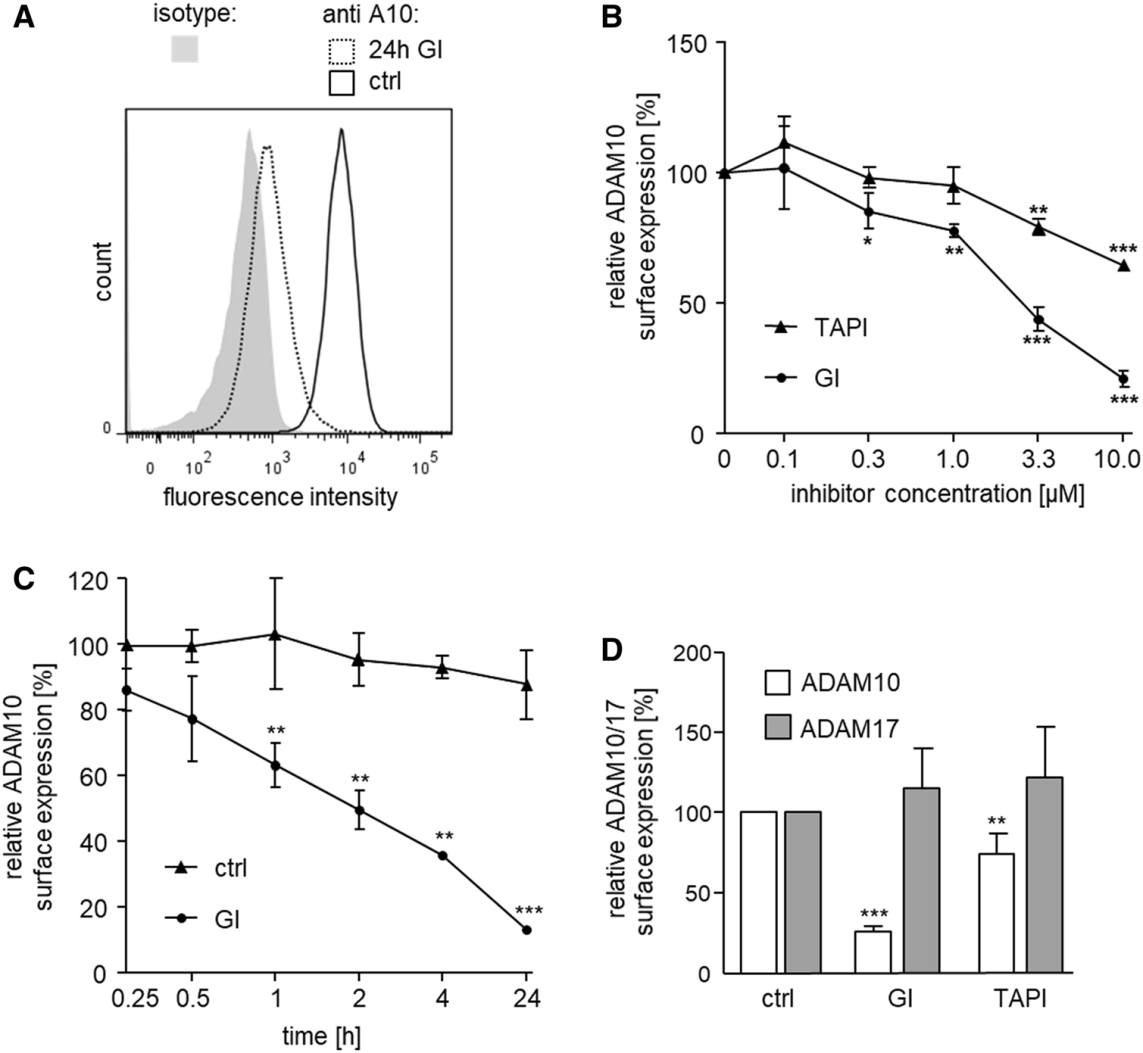
Effect of metalloproteinase inhibitors GI and TAPI on surface expression of ADAM10 and ADAM17**. a** THP-1 cells were treated with 10 μM GI, or DMSO as vehicle control (ctrl) for 24 h and subsequently analyzed for surface expression of ADAM10 by flow cytometry. Data are shown as representative histogram. **b** THP-1 cells were treated with the indicated concentrations of GI, TAPI or vehicle control for 24 h and subsequently analyzed for surface expression of ADAM10 (*n* = 3). **c** THP-1 cells were treated with 10 μM GI or vehicle control for the indicated periods of time and subsequently analyzed for surface expression of ADAM10 (*n* = 3). **d** THP-1 cells were treated with 10 μM GI, 10 μM TAPI or vehicle control for 24 h and subsequently analyzed for surface expression of ADAM10 (*n* = 7) or ADAM17 (*n* = 5). In **b**–**d,** the geometric mean fluorescence of inhibitor-treated cells was calculated in relation to that of the respective control and summarized as mean and SD of at least three independent experiments

Monocytic THP-1 cells were used to study the long-term effects of GI254023X on surface-expressed ADAM10. As demonstrated in detail in previous work, the cells express functional ADAM10 and the protease can be detected on the cell surface by staining with a monoclonal antibody against the extracellular N-terminus of ADAM10 and subsequent flow cytometric analysis [28]. We noted that a 24-h exposure of THP-1 cells to GI resulted in a considerable reduction of surface-detectable GI compared to control cells treated with vehicle (Fig. 1a). In further experiments, we verified that this effect was time- and concentration-dependent reaching about 15% residual ADAM10 expression after 24 h treatment with 10 µM GI (Fig. 1b, c). Of note, 15 min preincubation, which is enough for optimal inhibition of ADAM10 [21], is not sufficient for downregulation of ADAM10. Moreover, the long-term effect of GI was limited to ADAM10 and not seen for ADAM17 which is not a primary target of the inhibitor (Fig. 1b, d). We then tested TAPI-1 as a potent inhibitor of ADAM17 with additional inhibitory activity on ADAM10 (Fig. 1b, d). This inhibitor also did not affect ADAM17 surface detection. For ADAM10, some downregulation by TAPI-1 could be observed but not to the same extent as observed by GI treatment (Fig. 1b, d).

We next studied other cell types and found that GI also downregulates surface-detectable ADAM10 in epithelial lung carcinoma A549 cells without affecting ADAM17 (suppl. Fig. 1AB). Similarly, we observed GI-induced reduction of surface-detectable ADAM10 expression in HEK293 cells and primary human neutrophils (suppl. Fig. 1CD). Furthermore, another recently developed ADAM10 inhibitor (MN8) [33] significantly reduced the surface-detectable ADAM10 in THP-1 cells as well (suppl. Fig. 1E). Notably, there was neither an effect of GI on the mRNA expression of ADAM10 in THP-1 cells nor in A549 cells suggesting that ADAM10 seems to be regulated on a posttranslational level rather than on a transcriptional level (suppl. Fig. 1FG).

### GI254023X reduces total cellular content of mature ADAM10

**Fig. 2:**
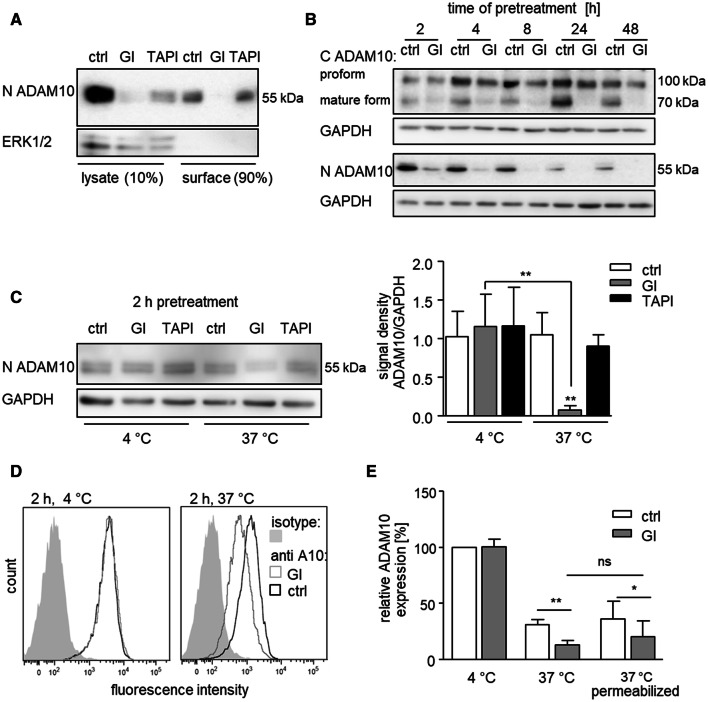
Effect of GI on ADAM10 in surface precipitates and cell lysates and temperature dependence of ADAM10 downregulation. **a** THP-1 cells were treated with 10 μM GI, 10 μM TAPI or vehicle control for 4 h. Surface proteins were biotinylated on intact cells and subsequently precipitated from cell lysates. Precipitates and lysates were then probed by western blotting with an antibody against the N-terminus of ADAM10. Detection of total cytosolic ERK1/2 served as a control. **b** THP-1 cells were treated with 10 μM GI or vehicle control for the indicated periods of time. Cells were lysed and lysates were then probed by western blotting with antibodies against the N-terminus or C-terminus of ADAM10 or against GAPDH as loading control. **c** THP-1 cells were treated with 10 μM GI, 10 μM TAPI or vehicle control for 2 h at either 4 °C or 37 °C. Cells were cooled on ice, lysed, and probed by western blotting with antibodies against the N-terminus of ADAM10 or against GAPDH as loading control. Data are shown as representative western blot and as relative changes of band intensity determined by densitometric analysis. **a–c** One representative western blot out of three independent experiments. **d–e** THP-1 cells were first stained with primary antibody against ADAM10, then treated with 10 μM GI or vehicle control for 2 h at either 4 °C or 37 °C and afterwards incubated with the secondary antibody on ice. Cells were either left intact or permeabilized before detection of ADAM10 by flow cytometry. Data were obtained in five independent experiments and are shown as representative histogram (**d**) or as means and SD of the geometric mean fluorescence in relation to that of the 4 °C control (**e**)

It is either possible that GI reduces the number of ADAM10 molecules on the cell surface or, alternatively, the inhibitor could simply prevent detection of the protease. To address this question, ADAM10 expression was studied by western blotting of cell lysates. In general, ADAM10 can be detected by antibodies against the extracellular N-terminus or antibodies against the intracellular C-terminus. The binding sites of these antibodies do not overlap with the protease’s active site. In comparison, the small molecule inhibitor GI254023X binds to the active site of ADAM10 and bigger natural inhibitors like TIMP-1 bind the active site and other sites as well (suppl. Fig. 2B) For western blot analysis, we first used the same N-terminal antibody against ADAM10 as for flow cytometry. This antibody detects two close protein bands with only small difference in molecular weight in lysates of THP-1 cells [31]. The specificity of this detection was controlled by shRNA-mediated knockdown of ADAM10 leading to disappearance of both bands (suppl. Fig. 2A). THP-1 cells were then exposed to GI and either lysed or used for biotinylation and precipitation of surface proteins. A clear reduction of ADAM10 protein in the lysate and in the surface precipitate was observed (Fig. 2a, suppl. Fig. 2C). By contrast, treatment with TAPI-1 led to a much weaker reduction of ADAM10 in the THP-1 cell lysate. The findings could be confirmed with A549 cells showing a predominant reduction of ADAM10 in surface precipitates after GI treatment and only a moderate reduction after TAPI-1 treatment (suppl. Fig. 2D).

Since the electrophoresis had to be run under non-reducing conditions for detection by the N-terminal antibody, we could not exclude an influence of remaining inhibitor on the detection of ADAM10. Moreover, it remained unclear whether GI can also affect the proform of ADAM10 since the N-terminal antibody only detected mature ADAM10.

### GI254023X does not downregulate the proform of ADAM10

To provide further evidence for downregulation of ADAM10, we used an antibody against the intracellular C-terminus of ADAM10 suitable for western blot detection under reducing conditions. Under these conditions, the structure would be completely destroyed and it is unlikely that non-covalently bound inhibitor would remain associated with the protease. Furthermore, this antibody is capable of detecting the proform as well as the mature form of ADAM10 as reported by us and others in several previous studies [21, 23]. The specificity of this detection was again shown by shRNA-mediated knockdown of ADAM10 resulting in complete loss of both protein bands (suppl. Fig. 2A).

It was shown recently that GI prevents C-terminal auto-degradation of ADAM10 as an artefact of cell lysis [34]. Therefore, we always added GI to our lysis buffer which is especially needed when using the C-terminal ADAM10 antibody. Consistent with previous reports, surface biotinylation showed that the pool of surface proteins contained both the pro- and the mature form of ADAM10 but only the mature form of ADAM17 (suppl. Fig. 2G) [13, 31, 35]. Both the C- and the N-terminal antibody detected the same time-dependent decrease of mature ADAM10 in the lysates after GI pretreatment (Fig. 2b, suppl. Fig. 2EF). Moreover, the proform of ADAM10 that could only be detected by the C-terminal antibody was not affected by GI. In summary, it can be concluded from our experiments that GI pretreatment of intact cells results in a loss of total mature ADAM10 but not the inactive proform.

### GI-induced ADAM10 downregulation is temperature dependent

We then asked whether the loss of mature ADAM10 would be temperature dependent. For this, cells were exposed to GI at 4 and 37 °C, lysed and studied by western blotting with the antibody against the N-terminus of ADAM10. We clearly noted that GI treatment only caused a loss of mature ADAM10 when the pretreatment was performed at 37 °C but not at 4 °C (Fig. 2c). These data suggest that GI-induced downregulation of mature ADAM10 is indeed temperature dependent.

We then studied GI-induced downregulation of surface-expressed ADAM10 on intact THP-1 cells by flow cytometry. Here, cells were incubated with the primary antibody against the N-terminus of ADAM10 at 4 °C prior to GI treatment. When subsequent GI treatment was performed at 4 °C, no effect on the ADAM10 surface level was observed (Fig. 2d, e, suppl. Fig. 2H). Compared to the 4 °C treatment, we noted a general reduction of surface-expressed ADAM10 even in the absence of GI when cells were preincubated at 37 °C and this reduction was clearly more prominent when GI was present. When THP-1 cells were permeabilized to make intracellular ADAM10 available, the reduction of the ADAM10 signal after GI treatment at 37 °C was still significant (Fig. 2e). As a positive control, we used the antibody against the intracellular C-terminus of ADAM10. As expected, permeabilization of the cells increased the fluorescence signal when cells were incubated with this antibody (suppl. Fig. 2I). Of note, when comparing non-permeabilized with permeabilized cells after GI treatment, we did not observe a prominent increase in ADAM10 which could have resulted from accumulation of internalized protease after GI treatment (Fig. 2e).

### GI-induced ADAM10 downregulation coincides with ADAM10 release in extracellular vesicles

**Fig. 3:**
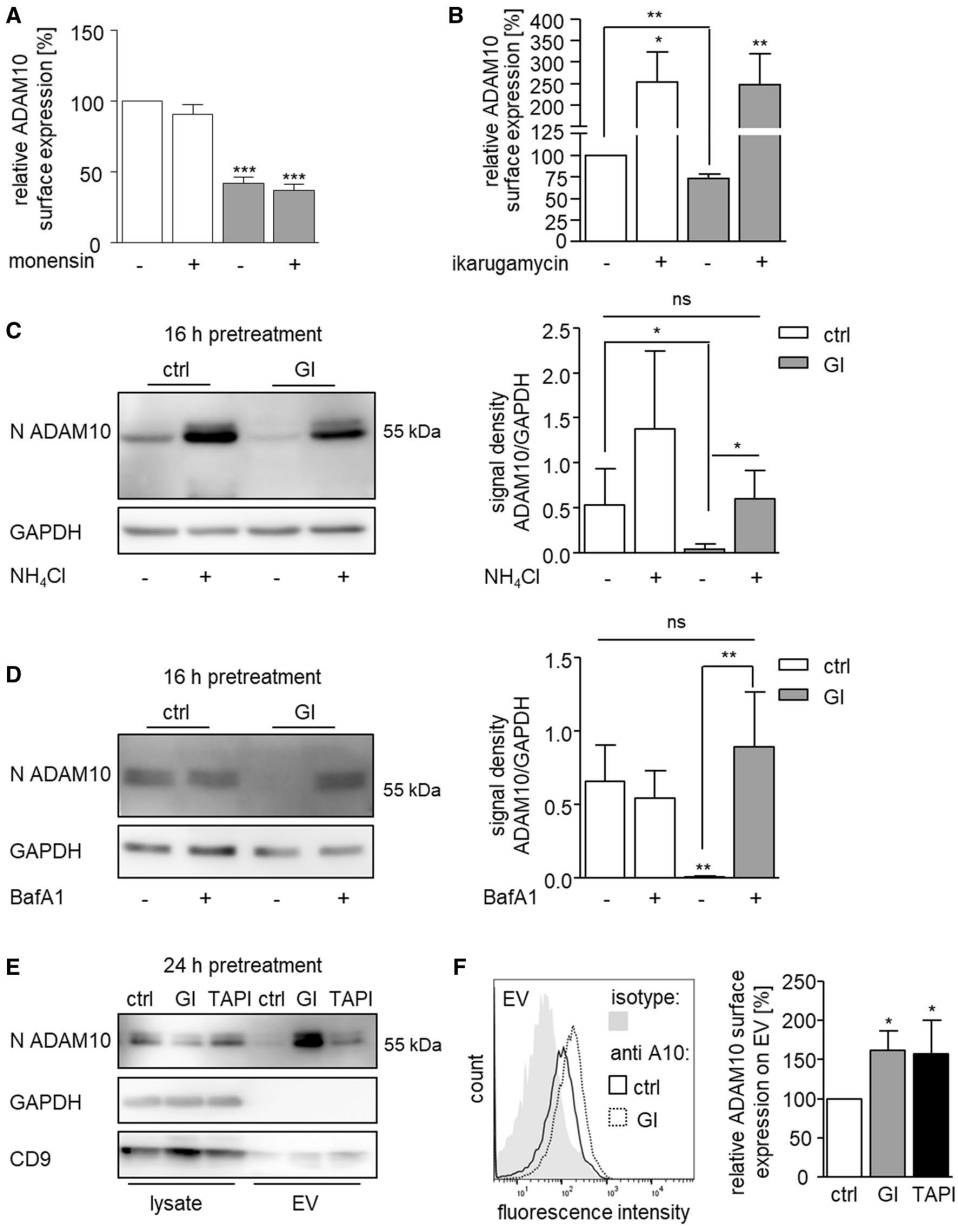
Effect of metalloproteinase inhibitors on ADAM10 internalization, degradation, and release in microvesicles. **a**, **b** THP-1 cells were treated with 10 μM GI or vehicle control. After 4 h (**a**) or 2 h (**b**), cells were analyzed for surface expression of ADAM10 by flow cytometry. The geometric mean fluorescence of treated cells was calculated in relation to that of the respective control and summarized as mean and SD of three independent experiments.) THP-1 cells were treated with 10 μM GI or vehicle control for 16 h in the absence or presence of 40 mM NH4Cl (**c**) or 0.5 μM bafilomycin A1 (**d**). Cell lysates were probed by western blotting with antibodies against the N-terminus of ADAM10 or against GAPDH as loading control. Data are shown as representative western blot and as relative changes of band intensity determined by densitometric analysis of three independent experiments. **e–f** THP-1 cells were treated with 10 μM GI, 10 μM TAPI or vehicle control for 24 h. Extracellular vesicles (EV) were prepared from conditioned cell media by differential centrifugation. Lysates and EV preparation were then subjected to western blot analysis with antibodies against the N-terminus of ADAM10, against the exosomal marker CD9 or against GAPDH as internal control (**e**). EV preparations from conditioned media were conjugated to beads which were then studied for ADAM10 immunoreactivity by flow cytometry (**f**). Data are shown as representative result or as means and SD of four independent experiments

We speculated that after GI treatment, ADAM10 may become internalized and subsequently degraded. When cells were treated with monensin which blocks protein forward trafficking, ADAM10 surface expression remained unaffected. However, additional GI treatment still induced downregulation of ADAM10 (Fig. 3a). Thus, an altered trafficking of ADAM10 from the Golgi to the cell surface appears unlikely. By contrast, the clathrin-mediated endocytosis inhibitor ikarugamycin caused a general increase of ADAM10 surface expression and prevented the GI-induced downregulation of ADAM10 from the cell surface (Fig. 3b). This finding confirms previous reports that ADAM10 undergoes constitutive internalization [13, 36] and also indicates that GI promotes internalization of ADAM10.

To study the potential involvement of lysosomal degradation, we treated cells with ammonium chloride (NH_4_Cl) or bafilomycin A1 which both block lysosomal acidification [37, 38]. Whereas bafilomycin A1 did not change the ADAM10 amount in the control cells, NH_4_Cl treatment clearly led to accumulation of ADAM10 in the lysates. Still, the surface expression of ADAM10 stayed unaltered under both agents (Fig. 3c, d, suppl. Fig. 3AB). Notably, ammonium chloride treatment partly rescued detection of ADAM10 in GI-treated cells which was even more pronounced when bafilomycin A1 was used. This indicates that the almost complete loss of ADAM10 due to GI treatment can be in part prevented by blocking lysosomal acidification.

Additionally, ADAM10 may be released in extracellular vesicles as shown in several previous studies [31, 39]. To investigate this possibility, we purified extracellular vesicles from THP-1 cells after GI or TAPI-1 treatment. In fact, loss of ADAM10 in the lysates correlated with a clear accumulation of ADAM10 in extracellular vesicles as detected by western blotting with either the N-terminal (Fig. 3e, suppl. Fig. 3C) or the C-terminal antibody (suppl. Fig. 3D). This increased release of vesicular ADAM10 was prominent with GI and much weaker with TAPI-1. To confirm these findings, isolated vesicles were coupled with beads which were then subjected to flow cytometry. Again, we observed significantly increased presence of ADAM10 on vesicles of those cells that were treated with the inhibitors (Fig. 3f).

### Recovery from GI-induced ADAM10 downregulation requires de novo synthesis

To investigate recovery from GI-induced ADAM10 downregulation, the inhibitor was removed by extensive washing followed by incubation of the cells in the absence of the inhibitor. While ADAM10 expression was profoundly reduced directly after inhibitor treatment, it was partly restored after 8 h and fully restored after 24 h of recovery from inhibitor treatment. This effect was seen for ADAM10 surface expression by flow cytometry as well as for total cellular ADAM10 expression by western blotting, whereas the transcriptional level of ADAM10 stayed unaltered (suppl. Fig. 4A–C).

**Fig. 4:**
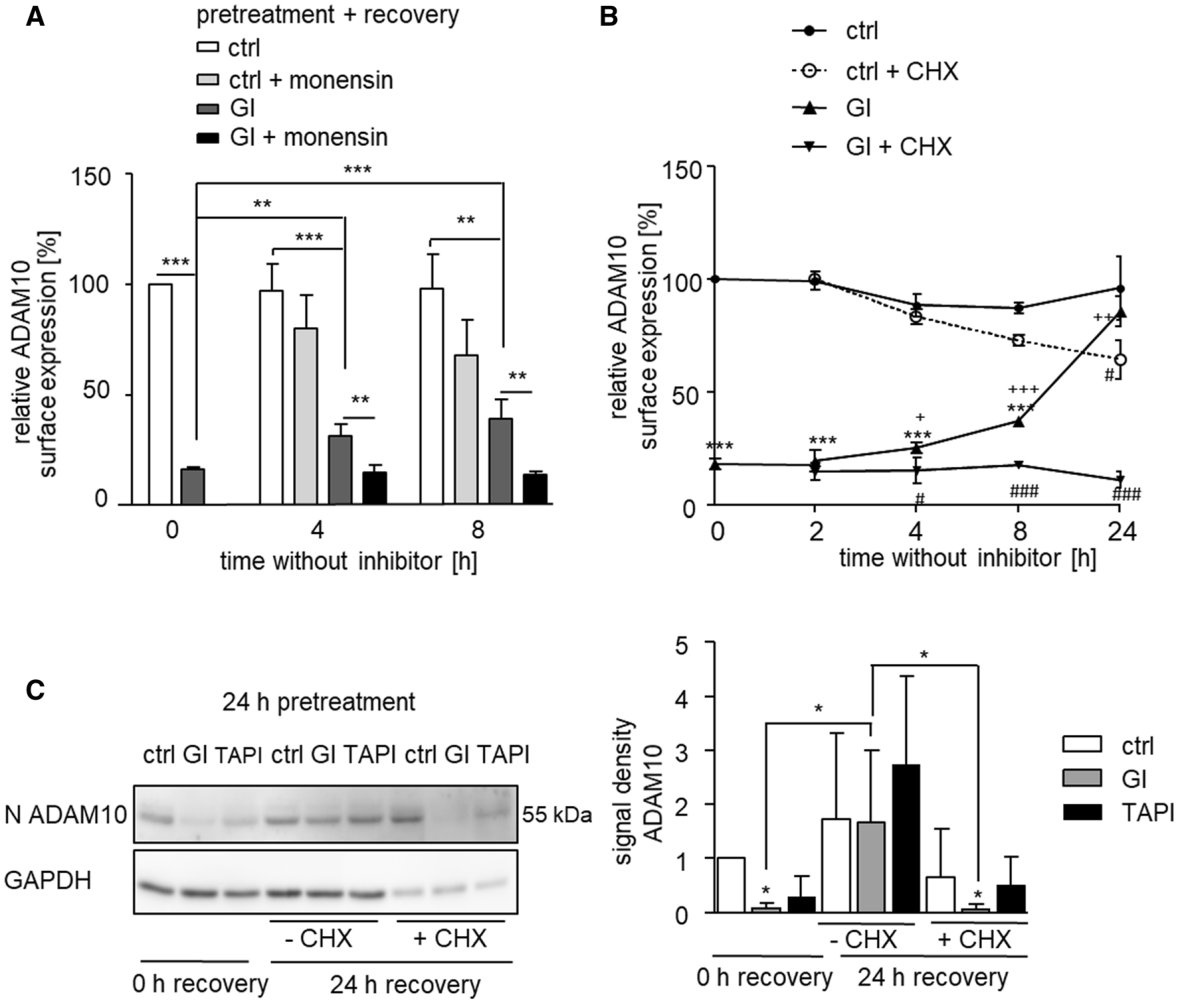
Recovery from ADAM10 depletion by de novo synthesis. **a** THP-1 cells were treated with 10 μM GI or vehicle control for 24 h. After washing, 2 μg/ml monensin or vehicle, control was added and cells were incubated for the indicated periods of recovery time before they were analyzed for surface expression of ADAM10 by flow cytometry. **b** THP-1 cells were treated with 10 μM GI or vehicle control for 24 h. After washing, 100 μM cycloheximide (CHX) or vehicle control was added and cells were incubated for the indicated periods of recovery time before they were analyzed for ADAM10 surface expression. ***p < 0.001 compared with ctrl at the respective time point, ^#^*p* < 0.05 comparison betwee* n* ± CHX, ^+^*p* < 0.05 compared with 0 h GI. **c** THP-1 cells were treated with 10 μM GI, 10 μM TAPI or vehicle control for 24 h. Washed cells were either further incubated with 100 μM cycloheximide (CHX) or vehicle control for 24 h to allow recovery or not further incubated. Subsequently, cells were lysed for western blot analysis with an antibody against the N-terminus of ADAM10 or against GAPDH as loading control. Relative changes of band intensity were determined by densitometric analysis. Data are shown as means and SD or as representative western blot of three independent experiments

In the next experiment, part of the samples received monensin during the recovery period to prevent transport of ADAM10 from the Golgi network through the secretory pathway to the cell membrane. Monensin treatment clearly prevented the recovery of ADAM10 surface expression from GI treatment (Fig. 4a). By contrast, monensin had no significant effect on ADAM10 expression of control cells not treated with GI.

In a similar experiment, cycloheximide was used to block de novo synthesis of ADAM10. In the absence of cycloheximide, we observed full restoration of ADAM10 surface expression after 24 h of recovery from GI treatment (Fig. 4b). This recovery was completely prevented by cycloheximide. Worth mentioning, 24 h cycloheximide treatment resulted in a reduction of the ADAM10 levels also in the control cells, showing the inhibition of the normal continual de novo synthesis of ADAM10. These findings were confirmed by western blot analysis of ADAM10 expression in cell lysates with the N-terminal antibody (Fig. 4c).

### Downregulation of ADAM10 is associated with impaired cellular shedding activity

**Fig. 5:**
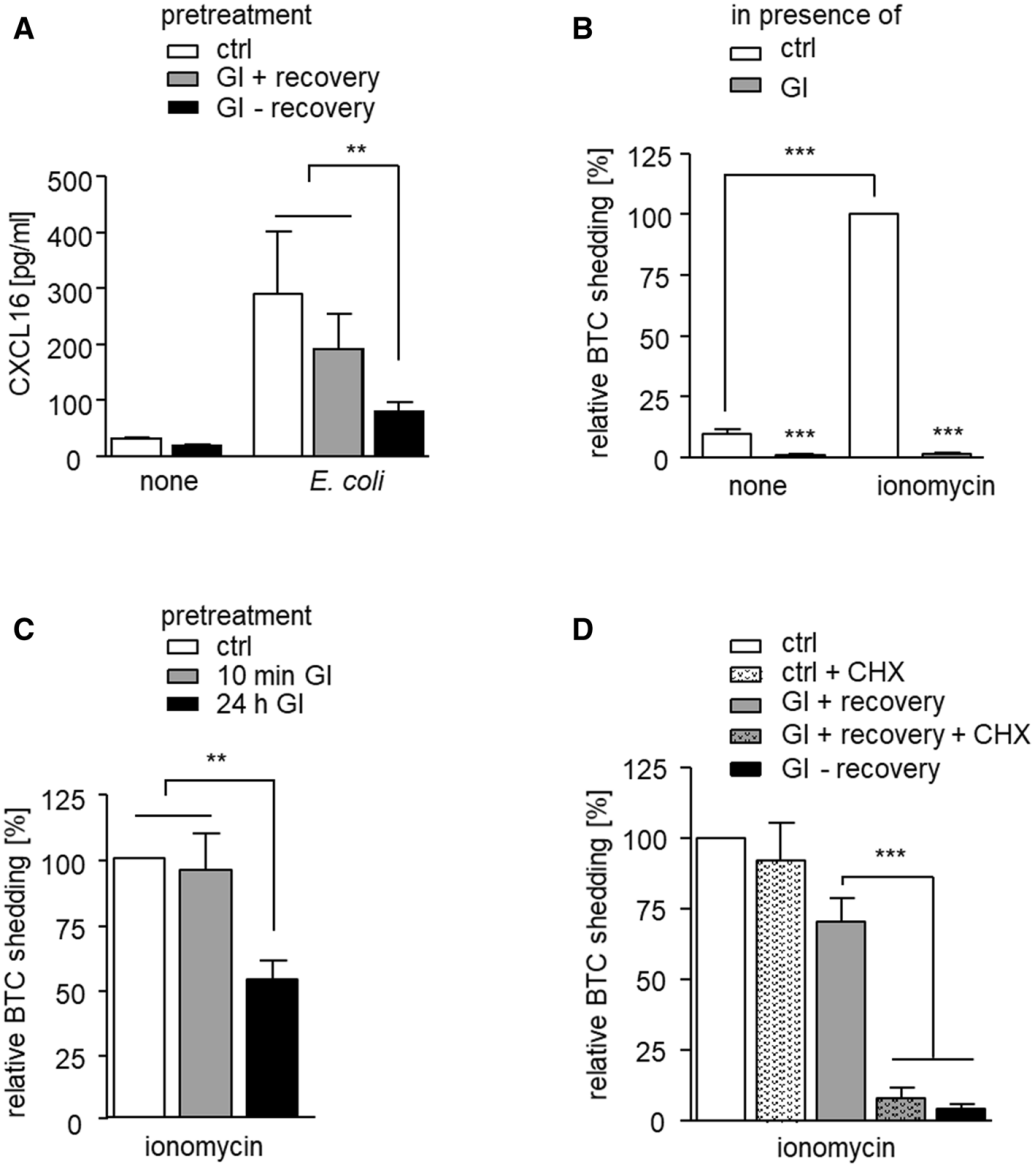
Loss and recovery of shedding activity after ADAM10 depletion by GI. **a** THP-1 cells were incubated with 10 μM GI or vehicle control and treated with pHrodo *E. coli* particles to induce CXCL16 expression or left untreated for 24 h. After washing, cells were incubated for another 24 h with pHrodo *E. coli* particles in the presence or absence of inhibitor and released CXCL16 was quantified in the conditioned media by ELISA. **b** HEK293 cells transfected with a betacellulin (BTC) alkaline phosphatase (AP) fusion protein were pretreated with 10 μM GI or vehicle control for 30 min and then stimulated with 1 μM ionomycin to increase ADAM10 activity or left unstimulated. After 45 min, the ratio of released to cell expressed BTC was determined by means of an AP activity assay. **c** BTC-AP transfected HEK293 cells were incubated with 10 μM GI or vehicle control for 10 min or 24 h. The inhibitor was removed by washing and cells were studied for ionomycin-induced BTC release as in **b**. **d** BTC-AP transfected HEK293 cells were incubated with 10 μM GI or vehicle control for 24 h. Cells were washed and either further incubated for 24 h to recover from inhibitor treatment or not allowed to recover (CHX treatment), before they were studied for ionomycin-induced BTC shedding as in **b**. Data in **b–d** were normalized to the ionomycin treated control. Data are shown as means and SD of three independent experiments

We questioned whether the observed downregulation and restoration of ADAM10 expression after inhibitor treatment would have implications for ADAM10-mediated shedding events. As a substrate we chose CXCL16 that can be induced in THP-1 cells by co-culture with bacteria and is shed at least in part via ADAM10 [23]. In fact, after GI treatment without recovery, we observed a strong reduction of CXCL16 shedding as determined by measurement of soluble CXCL16 in the culture supernatant (Fig. 5a). However, when the cells were allowed to recover from GI treatment, the majority of CXCL16 shedding was restored. To confirm these findings for a different cell type and for another substrate, we performed a shedding assay for betacellulin with HEK293 cells. For this, AP-coupled betacellulin (BTC) was transfected into HEK293 cells and the fusion protein was detected in the lysates and supernatants by an AP-activity assay. As described before, ADAM10-mediated shedding of betacellulin can be induced by cell stimulation with ionomycin [32]. We confirmed that ionomycin-induced betacellulin shedding was almost completely abrogated when GI was present during the shedding assay (Fig. 5b). Next, we pretreated cells with GI and removed the inhibitor before the assay. GI pretreatment for only 10 min did not affect the shedding activity (Fig. 5c). A likely explanation of this result is that short-term binding of GI to ADAM10 is not sufficient to yield a long-lasting effect on ADAM10 activity since inhibition by GI is rapidly reversible when the inhibitor is removed. However, when the pretreatment was extended to 24 h, the shedding activity was clearly reduced (Fig. 5c). Thus, after downregulation of ADAM10 as a result of long-term GI exposure, shedding is suppressed. Finally, 24-h pretreatment with GI plus 24-h recovery restored betacellulin shedding to approximately 75% of the control (Fig. 5d). As we showed above, downregulation of ADAM10 after long-term exposure to GI can be reversed after 24 h by de novo synthesis of ADAM10 consequently leading to restoration of its shedding activity.

### Physiological relevance of ADAM10 downregulation

**Fig. 6:**
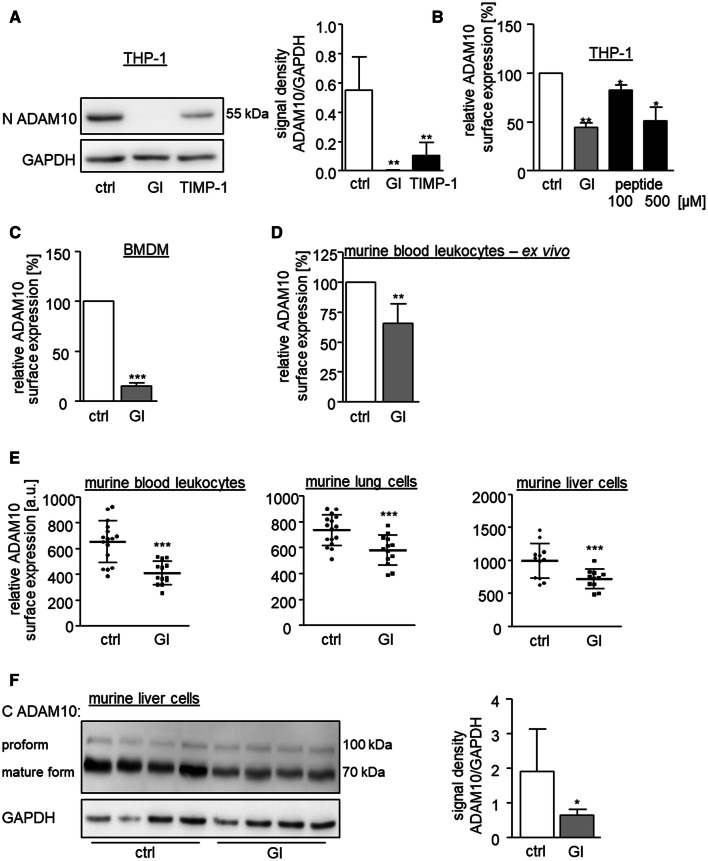
In vitro and in vivo relevance of ADAM10 downregulation. **a** THP-1 cells were treated with 200 nM recombinant TIMP-1, 10 μM GI or vehicle control for 24 h and subsequently studied for expression of mature ADAM10 by western blot analysis of cell lysates with antibodies against the N-terminus of ADAM10 and GAPDH as loading control. Data are shown as representative western blot and as relative changes of band intensity determined by densitometric analysis of three independent experiments. **b** THP-1 cells were treated with 10 μM GI, the indicated amounts of a specific ADAM10 peptide substrate or vehicle for 2 h and subsequently analyzed for surface expression of ADAM10 by flow cytometry. Geometric mean fluorescence intensity was calculated in relation to that of the untreated control (*n* = 3). **c** Isolated murine BMDMs were treated with 10 μM GI or vehicle control for 24 h and subsequently analyzed for surface expression of ADAM10 by flow cytometry. **d** Isolated murine peripheral blood leukocytes were treated with 10 μM GI, 10 μM TAPI or vehicle control for 2 h and subsequently analyzed for surface expression of ADAM10 by flow cytometry. In **c, d,** the geometric mean fluorescence of the cells was calculated in relation to that of the control and summarized as mean and SD of seven (**c**) or five (**d**) independent experiments. **e, f** Mice received daily intraperitoneal injections of 200 mg/kg bodyweight GI or DMSO as vehicle control. After 13 days, animals were sacrificed and ADAM10 surface expression on isolated peripheral blood leukocytes, lung or liver cells (**e**) was studied by flow cytometry. Data are shown as geometric mean fluorescence (*n* ≥ 11). **f** The western blot analysis of lysed liver tissue of four GI-treated and four control-treated mice with an antibody against the C-terminus of ADAM10 and GAPDH as loading control and the relative changes of band intensity of mature ADAM10 determined by densitometric analysis

We next used the metalloproteinase inhibitor TIMP-1 to study whether downregulation of ADAM10 can also occur with a natural inhibitor that is known to bind and inhibit ADAM10 [16, 21]. Recombinant human TIMP-1 was first tested for inhibition of ADAM10 activity in terms of betacellulin shedding. Pretreatment with a concentration of 200 nM TIMP-1 for 30 min led to considerable inhibition of ADAM10 activity but not as prominent as the inhibition by GI (suppl. Fig. 5A). Furthermore, treatment with 200 nM TIMP-1 for 24 h clearly reduced the presence of mature ADAM10 in cell lysates (Fig. 6a and suppl. Fig. 5B). Again, this effect was not as fast and as strong as that of GI. Finally, we questioned whether a high concentration of a described peptide substrate that binds to the active site of ADAM10 would cause oversaturation of the protease and thereby result in a similar downregulation of ADAM10. In fact, 24-h treatment with an optimized peptide [40] for ADAM10-mediated cleavage could diminish ADAM10 surface expression (Fig. 6b). These data clearly indicate that natural TIMP-1 and also high amounts of soluble substrate molecules can cause depletion of ADAM10. Thus, downregulation of ADAM10 is not restricted to synthetic inhibitors but represents a physiological phenomenon occurring with endogenous inhibitors and potentially also with high concentrations of soluble substrates.

Consequently, the next question we asked was whether GI-mediated downregulation of ADAM10 would be limited to human cells or also occur in mice. For this, we started experiments with the murine macrophage cell line RAW264.7 which showed GI-induced downregulation of ADAM10 after 24 to a comparable extent as seen for human cells (suppl. Fig. 5CD). Next, we wanted to test the GI effect in an ex vivo setting. Therefore, murine bone marrow-derived macrophages (BMDMs) were generated from femur and tibia (suppl. Fig. 5E) and murine leukocytes were prepared from retro-orbital blood samples of C57BL/6 mice. In both cases, GI treatment led to a clear downregulation of ADAM10 (Fig. 6c, d, suppl. Fig. 5F).

Having found that GI reduces ADAM10 in primary murine cells, we finally wanted to see if this effect is also present in vivo. Therefore, mice were treated with GI by repeated i.p. injection. As demonstrated by flow cytometry, ADAM10 surface expression was clearly reduced in murine leukocytes derived from peripheral blood and isolated lung and liver cells (Fig. 6e). Furthermore, the total cellular amount of ADAM10 was also decreased in the liver and lung cells (Fig. 6f, suppl. Fig. 5G).

**Fig. 7:**
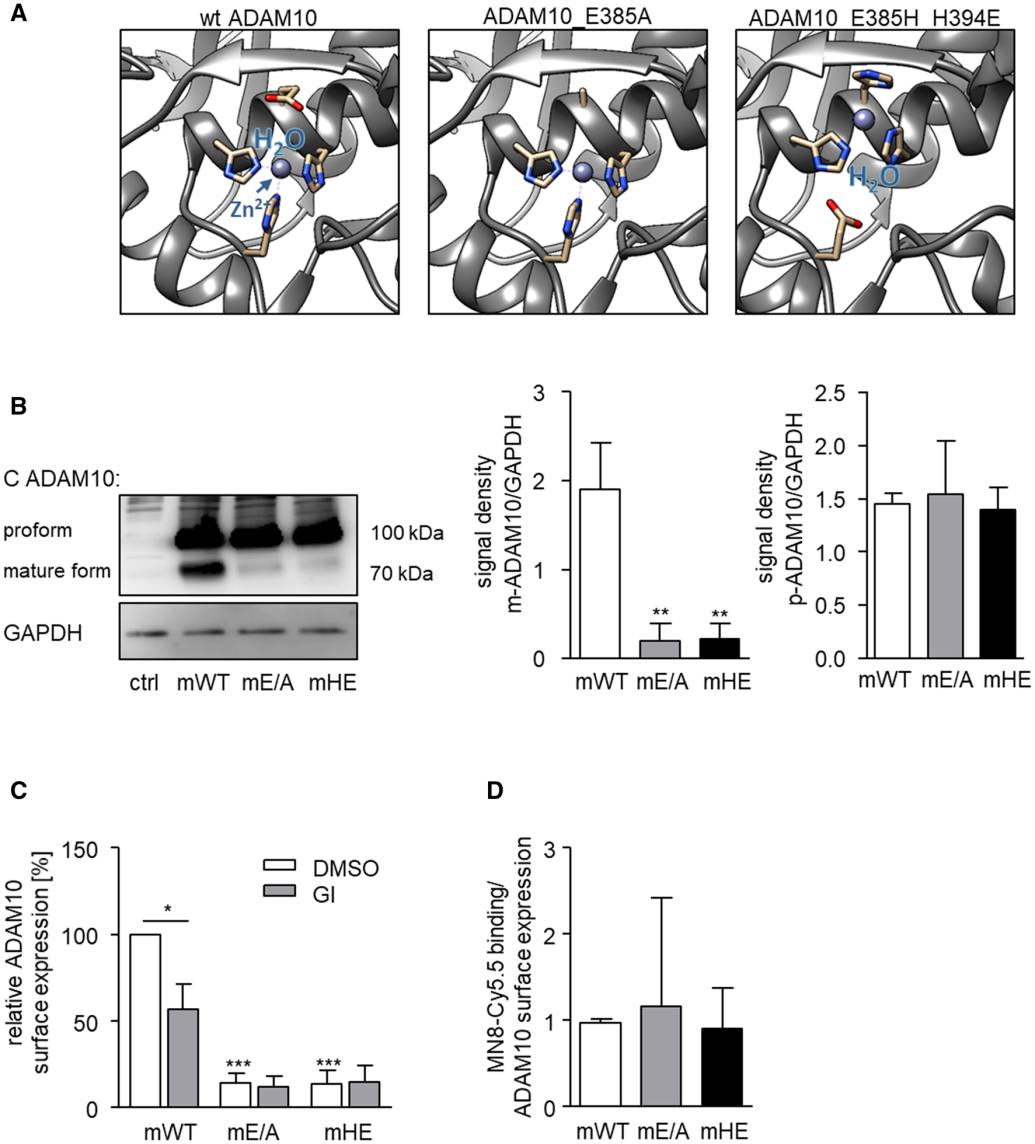
Expression and regulation of wild-type and inactive murine ADAM10 variants. **a** The active site within the catalytic domain of wt ADAM10 is depicted based on the ADAM10 structure pdb: 6be6 [19]. Here the three shown histidine residues coordinate the zinc ion (Zn^2+^), which together with the glutamate residue binds the water molecule needed for the hydrolysis of the peptide backbone of substrates. Therefore, substitution of the glutamate residue with an alanine residue (E385A) causes the loss of the protease activity. Changing the position of E385 with H394 results in a shifted active site which is not able to catalyse the hydrolysis of peptide bonds. **b–d** ADAM10-deficient HEK293 cells were transfected to express either murine WT-ADAM10 or the catalytically inactive murine ADAM10 mutants (mE/A-ADAM10 or mHE-ADAM10). Results are shown as representative western blot analysis of lysed HEK293 cells with an antibody against the C-terminus of ADAM10 and GAPDH as loading control (**b**). Relative changes in band intensity of mature and proform of ADAM10 were determined by densitometric analysis of three independent experiments. **c** Transfected cells were treated with 20 μM GI or vehicle control for 24 h and studied for murine ADAM10 surface expression. Results are shown as the geometric mean fluorescence of the ADAM10 expressing cells in relation to that of the control (murine WT-ADAM10 expressing cells) and summarized as mean and SD of three independent experiments. **d** Binding of Cy5.5-labeled MN8 to transfected HEK293 cells expressing ADAM10 variants was determined by flow cytometry. The specific fluorescence signal was calculated in relation to that of the ADAM10 surface expression determined for each variant (*n* = 3, see suppl. Fig. 6)

We then asked whether this ADAM10 downregulation occurs as a result of ADAM10 being inactive. To further investigate this, we introduced a single E to A amino acid residue replacement (E385A) at the active site of murine ADAM10 which is known to block the ADAM10 catalytic mechanism [41]. In another mutant, we switched the positions of H (H394E) and E (E385H) in the zinc-binding motif to disrupt the catalytic mechanism without changing the amino acid residue composition and the charges. Switching the glutamate residue with one of the histidine residues was earlier shown to abrogate protease activity of the structural-related metzincins MMP9 and ADAM17 without disrupting the structural integrity of their catalytic domain since interaction with TIMP-1 or TIMP-3, respectively, was not altered [42, 43]. Modeling of the introduced amino acid replacements showed that the E385A mutant lacked the glutamate for the catalytic mechanism and the zinc ion and water coordination may be affected, whereas in the H394E mutant, the zinc ion is displaced but its coordination with water should be unaffected [42, 43] (Fig. 7a). Importantly, these changes should not affect the substrate binding pockets or other parts of the metalloproteinase structure. These murine variants as well as murine wild-type ADAM10 were then expressed in a HEK293 cell line with knockout of endogenous ADAM10. Activity of wild-type ADAM10 and activity loss of the variants was confirmed by means of a betacellulin cleavage assay (suppl. Fig. 6A). Western blot analysis revealed that all variants were expressed at a comparable level with respect to their proforms. By contrast, the mature forms of the inactive variants were expressed at a much lower level compared to that of wild-type ADAM10 further demonstrating that inactivation of ADAM10 causes downregulation of its mature form (Fig. 7b). Moreover, GI treatment only reduced the expression of murine wild-type ADAM10, but did not further suppress the residual surface expression of the inactive variants (Fig. 7c). Nevertheless, at this stage, it could not be excluded that the mutants differ in their ability to bind the small molecule inhibitor which then might have led to differences in downregulation of the protease variants. We, therefore, used a Cy5.5-labelled derivate of the small molecule inhibitor MN8 against ADAM10. The inhibitor caused similar ADAM10 downregulation as GI254023X (compare suppl. Fig. 1E) and via its fluorescence, it also allows to investigate binding of the inhibitor to ADAM10. In fact, flow cytometric experiments indicated that the relative binding of the small molecule inhibitor was not compromised by introducing the mutations (Fig. 7d, suppl. Fig. 6BC). Thus, the observed differences in GI-induced downregulation of mature ADAM10 between the mutants and wild-type ADAM10 (Fig. 7c) were not due to differences in their inhibitor binding but rather differences in their proteolytic activity.

Taken together, these findings indicate that ADAM10 activity is required to maintain the full level of ADAM10 on the cell surface and that the observed downregulation could represent a physiological mechanism to remove the inactive protease.

## Discussion

In vitro and in vivo studies suggest that ADAM10 inhibition can suppress inflammatory and proliferative processes by shedding of inflammatory mediators or growth factors from the cell surface [1, 2, 44]. We here provide multiple lines of evidence that ADAM10 inhibitors not only block the active site of ADAM10 but also induce its downregulation. While inhibition of the active site is a rapid process occurring within minutes, we observed that downregulation of ADAM10 is temperature dependent and rather slow, requiring hours. This depletion is associated in parts with ADAM10 internalization and subsequent lysosomal degradation as well as with release of ADAM10 in extracellular vesicles. Full recovery from this depletion of ADAM10 takes several hours and requires de novo synthesis of ADAM10. Surface depletion of ADAM10 reduces shedding of the ADAM10 substrates CXCL16 and betacellulin. Consistent with the requirement of de novo synthesis after GI-mediated ADAM10 depletion, the recovery of the shedding activity is slow. We show that depletion of ADAM10 is not only an effect of synthetic inhibitors but also a physiological phenomenon also occurring with the natural inhibitor TIMP-1 and with overstraining the enzyme activity by oversaturating the protease with high amounts of substrate peptides. Moreover, inhibitor-mediated downregulation of ADAM10 can also be observed in vivo. Finally, downregulation is only observed with active ADAM10 but not with proteolytically inactive variants.

We found that long-term GI treatment of cells leads to downregulation of mature ADAM10 from the cell surface. This was evidenced by surface analysis of ADAM10 via flow cytometry as well as by surface precipitation of ADAM10 and western blot detection with an antibody against the N-terminus of ADAM10. Since the latter detection procedure was performed under denaturing conditions, it is very unlikely that GI remains bound to the protease and prevents the detection. In fact, GI not only reduces cell surface-expressed ADAM10 but also total mature ADAM10 as evidenced by western blotting with N- or C-terminus-targeting antibodies. We further show that this loss of ADAM10 occurs in many different cell types and is not seen for ADAM17 suggesting that it is truly an effect of the specific interaction of ADAM10 and its inhibitor.

The binding of either small synthetic inhibitors, the natural inhibitor TIMP-1 to the active site of ADAM10 or the oversaturation of the protease with substrate peptides can sufficiently induce downregulation of the protease. Since TIMP-1 is a large protein, it may cause surface downregulation of ADAM10 through a temperature-dependent mechanism, similar to that of an antibody when binding to a surface antigen. However, our results obtained with small molecule inhibitors or peptides, as well as the experiments with the inactive variants of ADAM10 strongly suggest that the inhibition of the shedding activity is responsible for the downregulation of the protease. When substrate cleavage is hindered by the inhibitors or by mutation of the catalytic site, the protease might remain associated with its substrate. This complex could then be removed possibly via regulatory mechanisms of the substrate. By such a mechanism, ADAM10 would be sorted together with its substrate for either internalization and subsequent recycling, lysosomal degradation or release in extracellular vesicles. This may also involve adapter molecules that interact with ADAM10 and regulate its surface expression such as the TspanC8 subfamily of tetraspanins [12, 45]. For instance, Tspan15, Tspan5, and Tspan14 differentially control ADAM10-mediated cleavage of distinct substrates [46, 47]. Further analysis of GI-mediated effects on these tetraspanins may provide new mechanistic insight into the natural regulatory mechanisms of ADAM10 surface expression and activity.

GI-induced ADAM10 downregulation only occurs at 37 °C indicating that cellular metabolism and function is required. In general, several mechanisms may account for temperature-dependent depletion of ADAM10. ADAM10 has been shown to be constitutively released in extracellular vesicles, while ADAM17 release occurs predominantly upon cell stimulation [15, 31]. Inhibitor treatment causes increased release of ADAM10 in extracellular vesicles as demonstrated by flow cytometry and by western blotting of isolated vesicles. Consistent with the surface downregulation of only the mature ADAM10 form, the vesicular ADAM10 had a molecular size corresponding to that of the mature form. It is unclear if this release in extracellular vesicles happens by direct budding of vesicles from the cell surface or if ADAM10 is internalized and afterwards released in extracellular vesicles. Besides this release in extracellular vesicles, other mechanisms may also contribute to surface downregulation of ADAM10. ADAM10 was found to be cleaved from the cell surface by ADAM15 and ADAM9 [14]. However, this would lead to the generation of cleavage fragments and in our experimental setup, no fragments were detected. ADAM10 may also be internalized and stored in vesicles [13, 48]. In fact, GI-induced downregulation of ADAM10 can be prevented with ikarugamcyin suggesting that ADAM10 undergoes clathrin-dependent internalization. Interestingly, we did not detect more ADAM10 when cells were permeabilized to make the intracellular pool of ADAM10 accessible. Moreover, our western blot analysis of the cell lysates demonstrates a clear loss of total mature ADAM10 upon GI treatment. This loss can be prevented by blocking lysosomal acidification with ammonium chloride or bafilomycin A1 indicating that internalized ADAM10 is degraded in the lysosomes.

Of note, the proform of ADAM10 which is thought be inactive is not regulated by GI although it can also be found at the cell surface [49]. Similarly, we found that the inactive ADAM10 variants can also be expressed at the cell surface and are insensitive to GI treatment. Different scenarios may be possible to explain these findings. Similar to the inactive ADAM10 variants, the proform of ADAM10 is constitutively transported to the cell surface where it is constitutively downregulated due to missing activity. However, the kinetic of this process would have to be in such a manner, that detecting immature ADAM10 on the cell surface is possible. Another putative explanation is that the ADAM10 proform is stable on the cell surface regardless of its catalytic inactivity because the presence of the prodomain might prevent downregulation by, for instance, hindering the binding to substrates.

The synthetic inhibitor GI has been used in several studies to block the activity of ADAM10 in cell culture experiments [17]. The inhibitor has also been administered in vivo to block shedding events in animal models of disease. For example, GI can prevent lung inflammatory processes and fibrosis development in lungs [10]. Additionally, ADAM10 can serve as a receptor for the *S. aureus* alpha toxin and GI was shown to suppress this toxicity in a murine model of *S. aureus* lung infection [11]. These findings indicate that treatment with GI could represent a therapeutic option especially in inflammatory, fibrotic, or infectious lung diseases. So far, it has been thought that the mode of action of GI would be limited to its inhibition of ADAM10 proteolytic activity. Our study demonstrates that GI causes downregulation of ADAM10 not only in an in vitro setting but also in leukocytes from peripheral blood and from liver and lung cells in vivo. This depletion of surface-expressed ADAM10 would not only affect metalloproteinase-related functions but also binding functions of the protease such as potential interaction with integrins or with bacterial toxins. When GI is removed from the system, it can be expected that inhibition will still persist for some time since ADAM10 has to be resynthesized to restore surface expression. This has to be considered when ADAM10 inhibition is no longer wanted or treatment cannot be continued due to side effects. Nevertheless, some recovery from downregulation can be observed after 24 h. In case further ADAM10 inhibition is necessary, it would be advisable to re-administer GI within this time frame.

Endogenous inhibitors such as TIMP-1 can control excess ADAM10 activity [16]. Our study shows that TIMP-1 or even an excess of ADAM10 substrate peptides that are derivatives of the regions of natural substrates, which harbor the ADAM10 cleavage sites, can induce downregulation of ADAM10 similar as seen for GI. This indicates that the regulation of ADAM10 is not only limited to synthetic compounds but also represents a physiological phenomenon. Taken together, we propose that this downregulation represents a natural mechanism to remove ADAM10 from the cell surface in a situation when endogenous or exogenous molecules impact the activity of the protease. Concurrently, bound inhibitors would also be removed as they would either undergo degradation or exosomal release together with inactivated ADAM10 and this would then allow to restore ADAM10 activity over time by de novo synthesis of the protease. Furthermore, one may speculate that other cell-surface proteases undergo a similar regulation, which would introduce a new aspect to consider, when analyzing these proteases in search for ways to therapeutically block their activity.

### Electronic supplementary material

Below is the link to the electronic supplementary material.

Supplementary file1 (PDF 733 kb)

## Abbreviations

ADAM: A disintegrin and metalloproteinase
AP: Alkaline phosphatase
BMDM: Bone marrow-derived macrophage
BTC: Betacellulin
CHX: Cycloheximide
ctrl: Control
DMEM: Dulbecco’s Modified Eagle’s Medium
EGF: Epidermal growth factor
ERK: Extracellular signal-regulated kinase
EV: Extracellular vesicle
FDR: False discovery rate
GAPDH: Glyceraldehyde 3-phosphate dehydrogenase
GI: GI254023X
HSP: Heat shock protein
i.p.: Intraperitoneal
shRNA: Small hairpin RNA
TAPI: TAPI-1
TIMP-1: Tissue inhibitor of metalloproteinase 1
WT: Wild type
