# Supplementary material for: The metalloproteinase ADAM10 requires its activity to sustain surface expression
Source: Cell Mol Life Sci. 2020 May 5;78(2):715–32. doi: 10.1007/s00018-020-03507-w (PMC7873107; doi:10.1007/s00018-020-03507-w)
Supplement: Supplementary file 1 — Supplementary file1 (PDF 733 kb) [file 18_2020_3507_MOESM1_ESM.pdf]

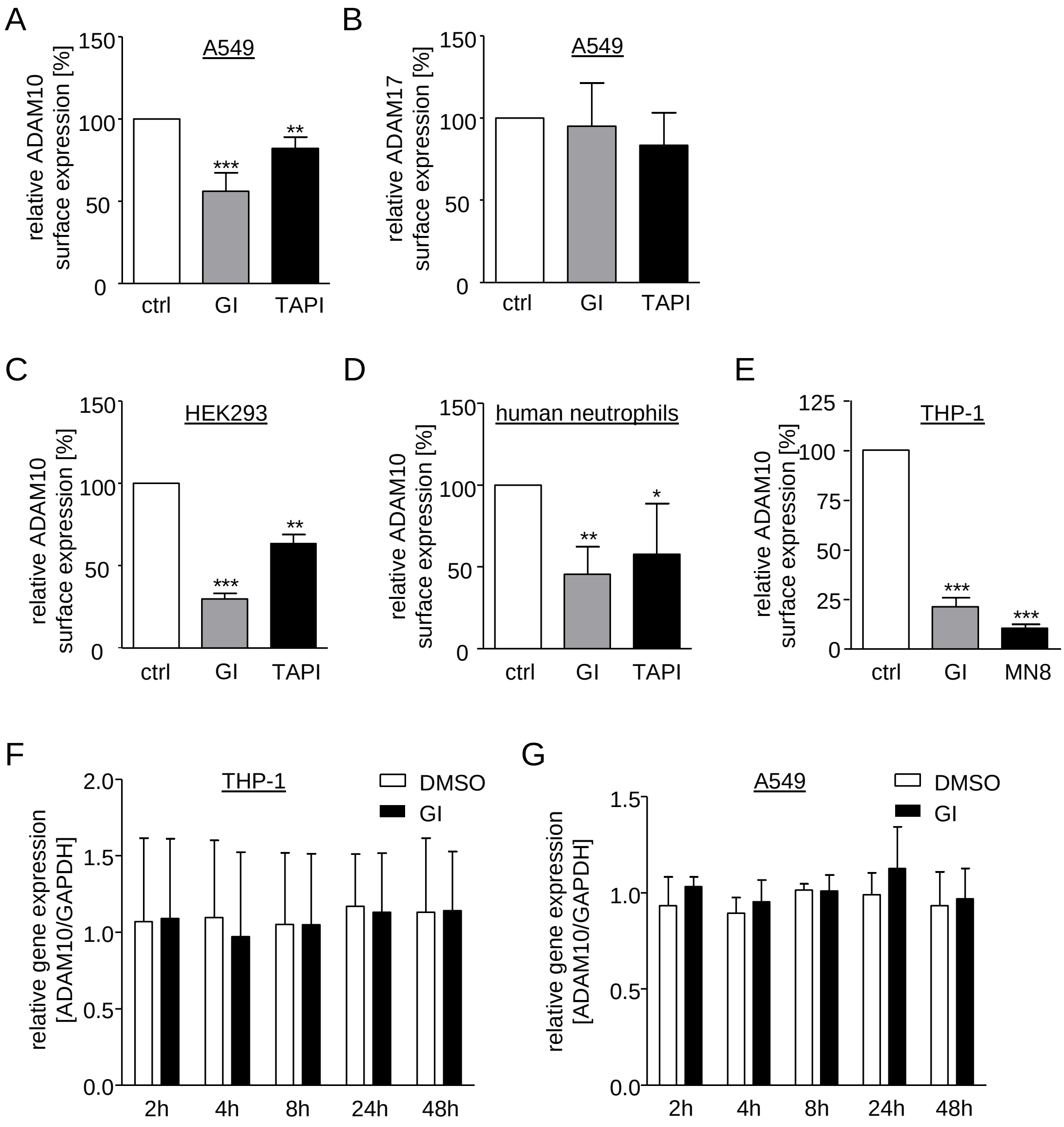

Supplemental Figure 1

A-B) A549 cells were treated with 10 µM GI, 10 µM TAPI or vehicle control for 24 h and subsequently analyzed for surface expression of ADAM10 (A) and ADAM17 (B) by flow cytometry. **C**) HEK293 cells were treated with 10 µM GI, 10 µM TAPI or vehicle control for 24 h and subsequently analyzed for surface expression of ADAM10. **D**) Human neutrophils from peripheral blood were treated with 10 µM GI, 10 µM TAPI or vehicle control for 24 h and subsequently surface expression of ADAM10 was investigated. **E**) THP-1 cells were treated with 10 µM GI, 10 µM MN8 or vehicle control for 24 h and subsequently analyzed for surface expression of ADAM10. In A-E geometric mean fluorescence intensity of inhibitor treated cells was calculated in relation to that of the control. **F, G**) THP-1 cells (F) or A549 cells (G) were treated with 10 µM GI or vehicle control for the indicated time periods and subsequently analyzed for mRNA expression of ADAM10 by RT-qPCR. All data represent means and SD of at least three independent experiments.

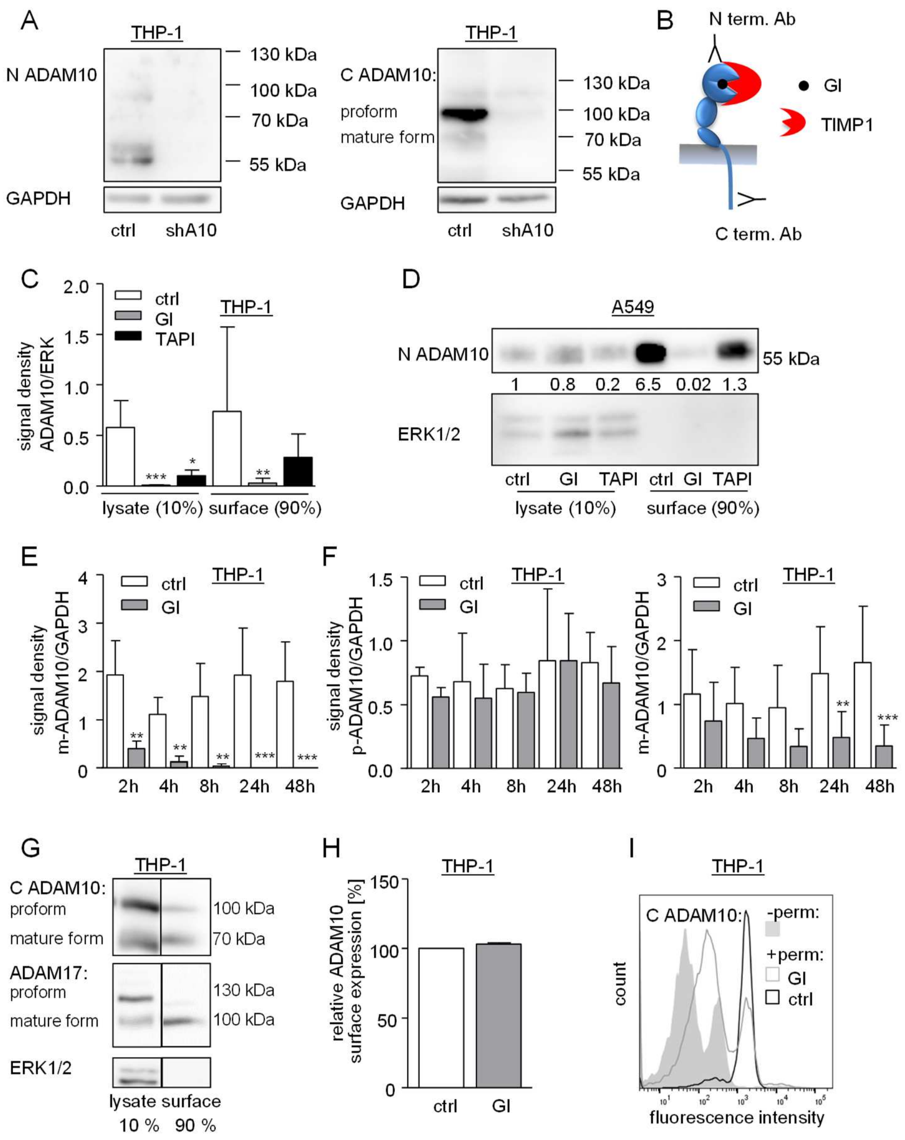

Supplemental Figure 2

A) THP-1 cells were transduced with lentivirus encoding shRNA against ADAM10 or irrelevant shRNA (ctrl). Transduced cells were lysed and probed by western blotting with antibodies against the N-terminus (left) or C-terminus (right) of ADAM10 or against GAPDH as loading control. **B**) Graphical model of ADAM10 showing the binding sites of the N- and C-terminal antibodies and the inhibitors GI and TIMP-1. **C**) Densitometric analysis of the band intensity of mature ADAM10 from Fig. 2A (n=3). **D**) A549 cells were treated with 10 µM GI, 10 µM TAPI or vehicle for 4 h. Surface proteins were biotinylated on intact cells and subsequently precipitated from cell lysates. Surface precipitates and lysates were then probed by western blotting with an antibody against the N-terminus of ADAM10. ERK1/2 was detected as control. Relative changes of band intensity determined by densitometric analysis are given as numbers below each band. **E-F**) Densitometric analysis of the band intensity of pro- and mature ADAM10 detected with antibodies against the ADAM10 N-terminus (E) or C-terminus (F) (compare Fig. 2B, n=3). **G**) Surface proteins of THP-1 cells were biotinylated on intact cells and subsequently precipitated from cell lysates. Precipitates and lysates were then probed by western blotting with antibodies against the C-terminus of ADAM10 or ADAM17. Detection of total cytosolic ERK1/2 served as a control. **H**) THP-1 cells were treated with 10 µM GI or vehicle control for 2 h at 4 °C, washed, stained and subsequently analyzed for surface expression of ADAM10 by flow cytometry. The geometric mean fluorescence intensity of inhibitor treated cells was calculated in relation to that of the control. Data represent means and SD of three independent experiments. **I**) THP-1 cells were treated with 10 µM GI or vehicle control for 2 h at 37 °. Cells were either left intact or permeabilized before detection of ADAM10 by flow cytometry using the antibody against the ADAM10 C-terminus. Data are shown as representative histogram.

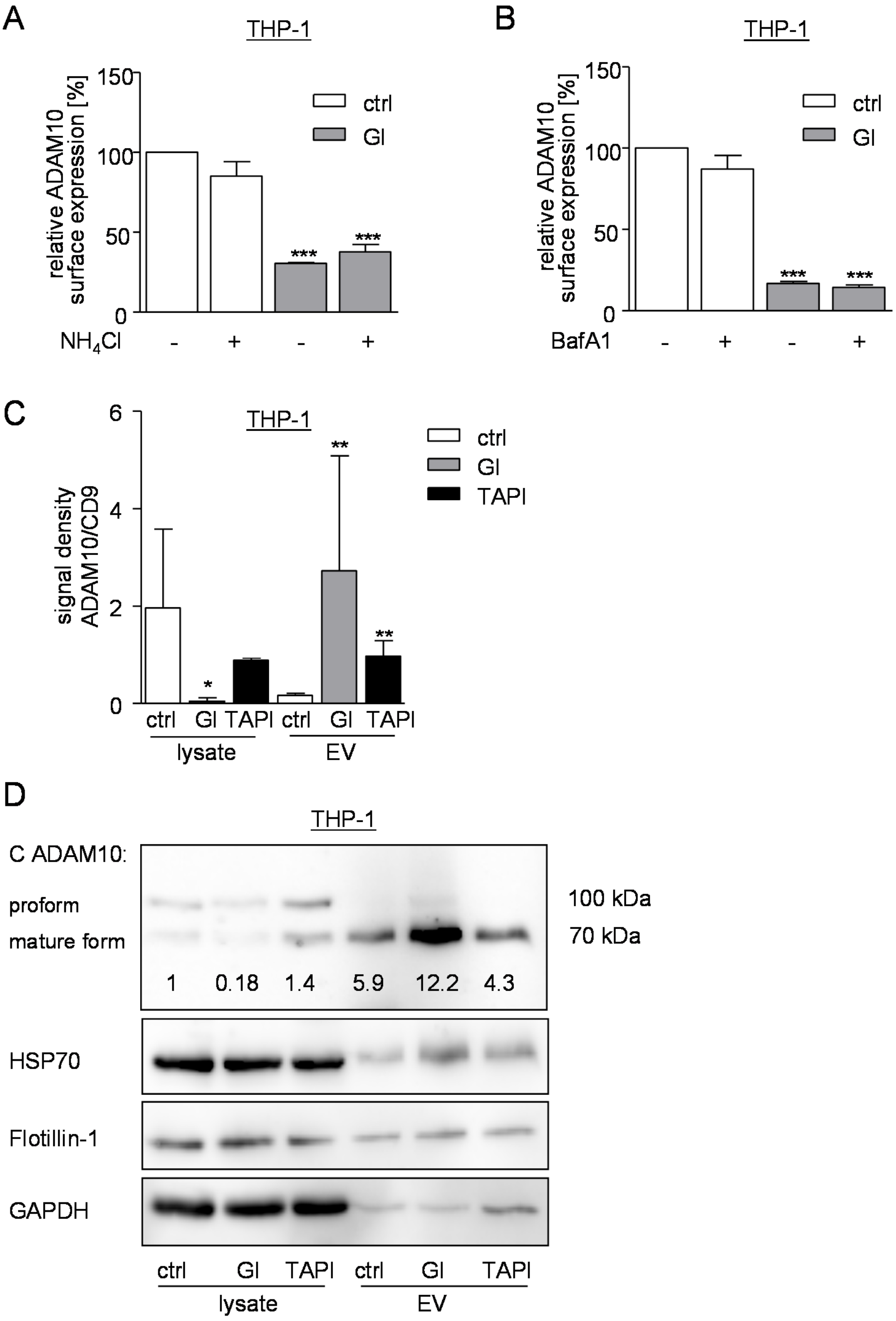

Supplemental Figure 3

A-B) THP-1 cells were treated with 10 µM GI or vehicle control for 16 h in the absence or presence of 40 mM NH_4Cl (A) or 0.5 µM bafilomycin A1 (B) and subsequently analyzed for surface expression of ADAM10 by flow cytometry. Geometric mean fluorescence intensity was calculated in relation to that of the untreated control. **C**) Densitometric analysis of the band intensity of mature ADAM10 from Fig. 3E .**D**) THP-1 cells were treated with 10 µM GI, 10 µM TAPI or vehicle control for 24 h. Extracellular vesicles (EV) were prepared from conditioned cell media by differential centrifugation. Lysates and EV preparation were then subjected to western blot analysis with antibodies against the C-terminus of ADAM10, against the exosomal markers HSP70 and Flotilin-1 and against GAPDH as control. Data are shown as mean and SD or as representative western blot of three independent experiments. Numbers below bands indicate relative changes of signal intensity determined by densitometric analysis.

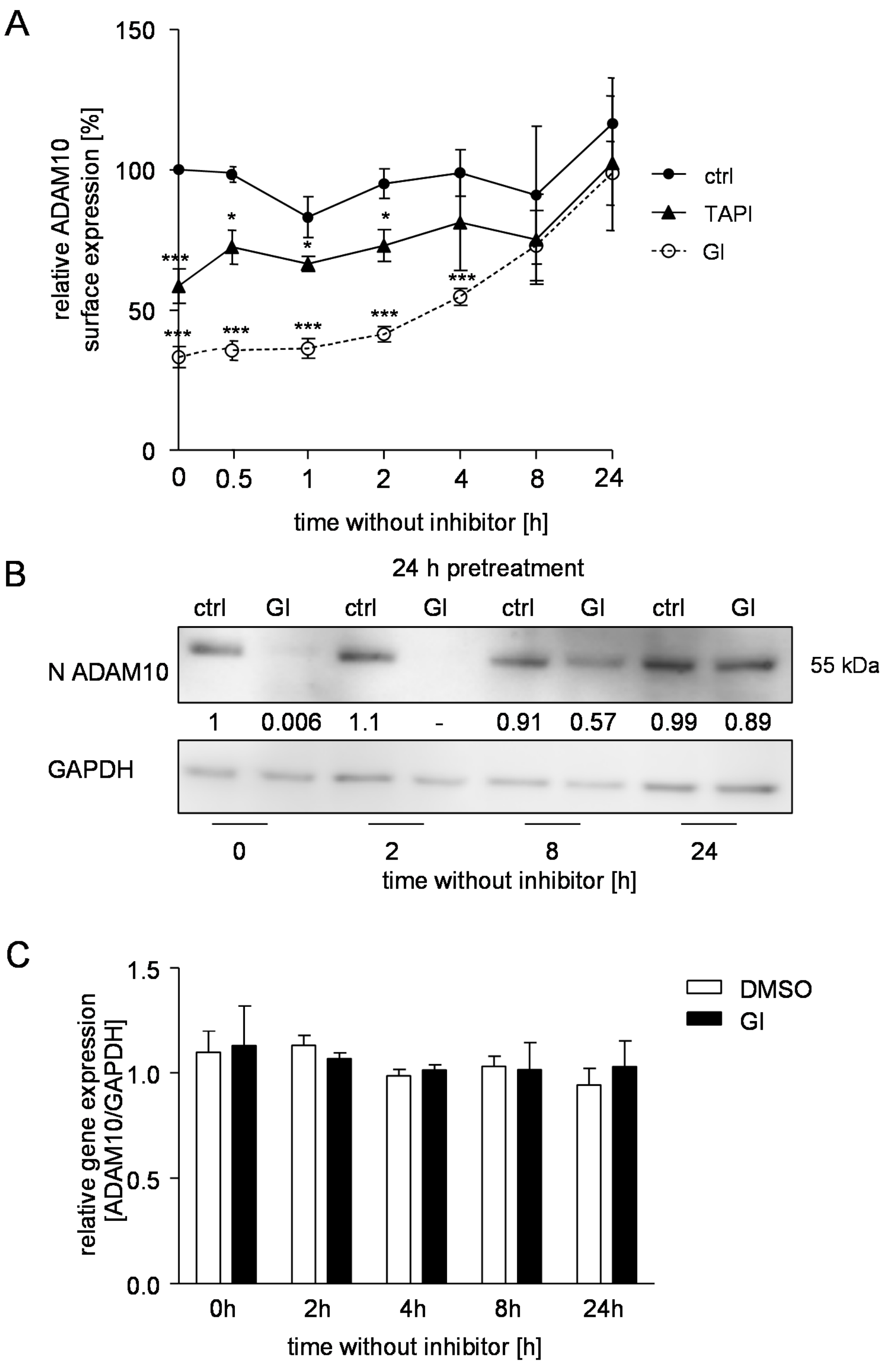

Supplemental Figure 4

A) THP-1 cells were treated with 10 µM GI, 10 µM TAPI or vehicle control for 24 h. After washing, cells were incubated for the indicated periods of time in the absence of inhibitor and analyzed for surface expression of ADAM10 by flow cytometry. The geometric mean fluorescence of inhibitor treated cells was calculated in relation to that of the control. **B, C**) THP-1 cells were treated with 10 µM GI or vehicle control for 24 h. Washed cells were incubated for the indicated periods of time in the absence of inhibitor. Subsequently cells were either lysed for western blot analysis with an antibody against the N-terminus of ADAM10 or against GAPDH as loading control (B) or analyzed for mRNA expression of ADAM10 by RT-qPCR (C). Data were obtained in three independent experiments and are shown as one representative result or as means and SD.

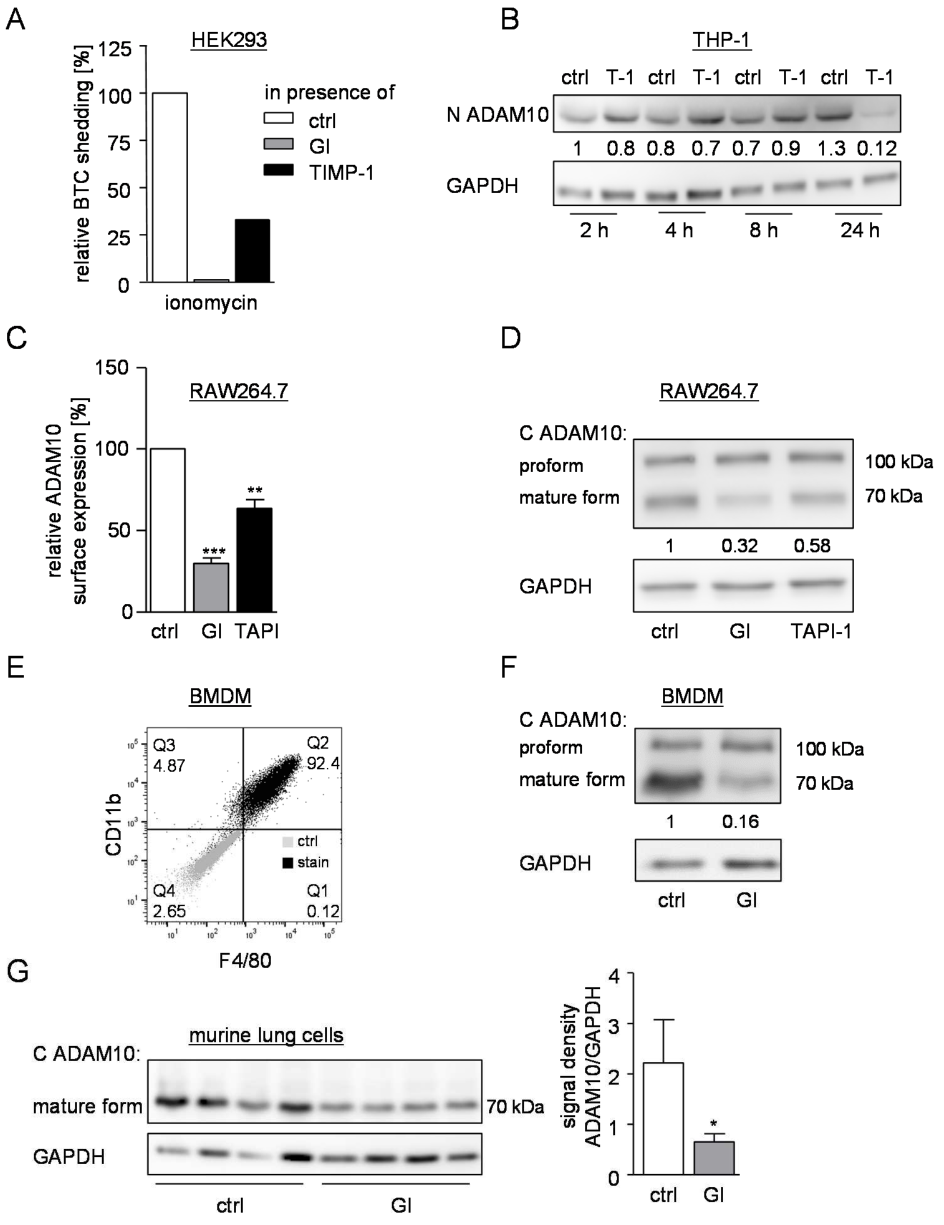

Supplemental Figure 5

A) HEK293 cells transfected with a BTC-AP fusion protein were pretreated with 10 µM GI, 200 nM TIMP-1 or vehicle control for 30 min and then stimulated with 1 µM ionomycin to increase ADAM10 activity or left unstimulated. After 45 min, the ratio of released to cell expressed BTC was determined by means of an AP activity assay (n=1). **B**) THP-1 cells were treated with 200 nM recombinant TIMP-1 or vehicle control for the indicated periods of time and subsequently studied for expression of mature ADAM10 by western blot analysis of cell lysates with an antibody against the N-terminus of ADAM10 or GAPDH as loading control. Numbers below bands indicate changes of signal intensity determined by densitometric analysis. **C, D**) RAW264.7 cells were treated with 10 µM GI, 10 µM TAPI or vehicle for 24 h and subsequently analyzed for surface expression of ADAM10 by flow cytometry (C) or cells were lysed and then probed by western blotting with antibodies against the C-terminus of ADAM10 and GAPDH as control (D) (n=3). Indicated percentages show relative changes of band intensity determined by densitometric analysis. **E**) Isolated BMDMs were stained with the macrophage markers F4/80 and CD11b and analyzed by flow cytometry. **F**) BMDMs were treated with 10 µM GI or vehicle control for 24 h, cells were lysed and probed by western blotting with antibodies against the C-terminus of ADAM10 and GAPDH as loading control. Indicated percentages show relative changes of band intensity determined by densitometric analysis. **G**) Western blot analysis of lysed lung tissue of four GI treated and four control mice with an antibody against the C-terminus of ADAM10 and the relative changes of band intensity of mature ADAM10 determined by densitometric analysis.

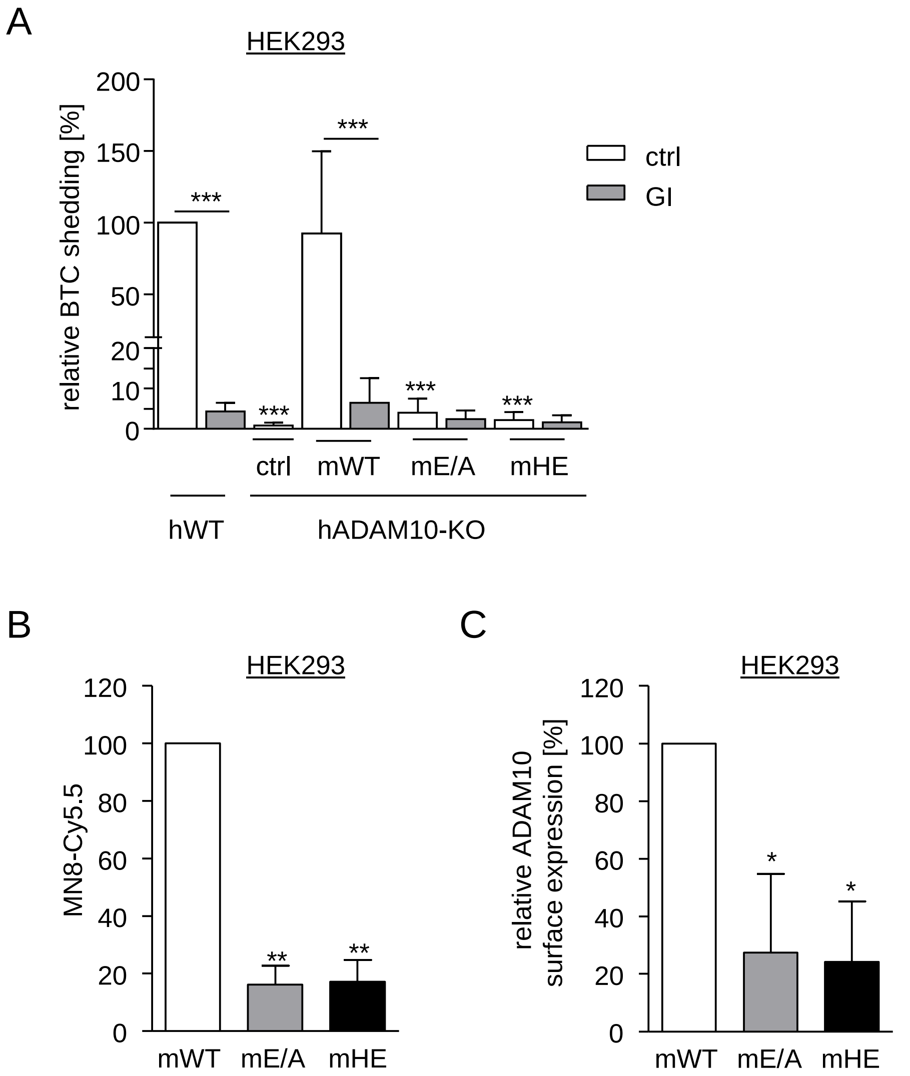

Supplemental Figure 6

A) ADAM10 deficient HEK293 cells (hADAM10-KO) were cotransfected with a BTC-AP fusion protein and either murine WT-ADAM10 (mA10) or the ADAM10 mutants (mE/A-ADAM10 or mHE-ADAM10). As controls normal BTC-AP transfected HEK293 cells and ADAM10 deficient HEK293 cells were used. The transfected HEK293 cells were pretreated with 10 µM GI or vehicle control for 30 min and then stimulated with 1 µM ionomycin. After 45 min, the ratio of released to cell expressed BTC was determined using an AP activity assay. **B-C**) ADAM10-deficient HEK293 cells were transfected to express either murine WT-ADAM10 or the catalytically inactive murine ADAM10 mutants (mE/A-ADAM10 or mHE-ADAM10). Transfected cells were studied either for specific binding of Cy5.5 labeled MN8 (B) or for murine ADAM10 surface expression (C) by flow cytometry. Results are shown as the geometric mean fluorescence of the transfected cells in relation to that of murine WT-ADAM10 expressing cells. Data are shown as mean and SD of three independent experiments.
